# Genome-wide analysis of *Enterococcus faecalis* genes that facilitate interspecies competition with *Lactobacillus crispatus*

**DOI:** 10.1128/jb.00438-24

**Published:** 2025-02-04

**Authors:** Ling Ning Lam, Kathryn E. Savage, Camille N. Shakir, José A. Lemos

**Affiliations:** 1 Department of Oral Biology, College of Dentistry, University of Florida 164889 , Gainesville, Florida, USA; University of Illinois Chicago, Chicago, Illinois, USA

**Keywords:** enterococci, lactobacilli, *E. faecalis*, interspecies competition, bacterial antagonism

## Abstract

Enterococci are opportunistic pathogens notorious for causing a variety of infections. While both *Enterococcus faecalis* and *Lactobacillus crispatus* are commensal residents of the vaginal tract, the molecular mechanisms that enable *E. faecalis* to take advantage of a vaginal biome with lower counts of lactobacilli to colonize the vaginal tract and induce aerobic vaginitis remain unknown. Here, we show that *L. crispatus* eradicates *E. faecalis* in a contact-independent manner. Using transposon sequencing to identify *E. faecalis* OG1RF transposon (Tn) mutants that are either under-represented or over-represented when co-cultured with *L. crispatus*, we found that Tn mutants with disruption in the *dltABCD* operon, that encodes the proteins responsible for the D-alanylation of teichoic acids, and *OG1RF_11697* encoding for an uncharacterized hypothetical protein are more susceptible to killing by *L. crispatus*. Inversely, Tn mutants with disruption in *ldh1,* which encodes for L-lactate dehydrogenase, are more resistant to *L. crispatu*s killing. Using the *Galleria mellonella* infection model, we show that co-injection of *L. crispatus* with *E. faecalis* OG1RF enhances larvae survival while this *L. crispatus*-mediated protection was lost in larvae co-infected with either *L. crispatus* and *E. faecalis*Δ*ldh1* or Δ*ldh1*Δ*ldh2* strains. Last, using RNA sequencing to identify *E. faecalis* genes that are differently expressed in the presence of *L. crispatus*, we found major changes in the expression of genes associated with glycerophospholipid metabolism, central metabolism, and general stress responses. The findings in this study provide insights into how *E. faecalis* mitigate assaults by *L. crispatus*.

**IMPORTANCE:**

*Enterococcus faecalis* is an opportunistic pathogen notorious for causing a multitude of infections. As vaginal commensals, *E. faecalis* must interact with *Lactobacillus crispatus*, but how *E. faecalis* overcomes or mitigate assaults by *L. crispatus* killing remains unknown. We show that *L. crispatus* eradicates *E. faecalis* temporally in a contact-independent manner. Using high-throughput molecular approaches, we identified genetic determinants that enable *E. faecalis* to compete with *L. crispatus*. This study represents an important first step for the identification of adaptive genetic traits required for enterococci to tolerate assaults by lactobacilli.

## INTRODUCTION

Enterococci, residents of the gastrointestinal and urogenital tracts, are notorious for causing a variety of opportunistic infections including but not limited to catheter-associated urinary tract infections (CAUTI) (1), antibiotic-induced intestinal dysbiosis (2, 3), surgical site and diabetes-associated wound infections (4), endodontic infections (5), and infective endocarditis (IE) (6, 7). Notably, enterococci are among the top five bacterial pathogens associated with bloodstream infections, surgical site infections, and CAUTI (8, 9), and are the third most common bacterial agent of IE (10). Armed with the ability to acquire antibiotic resistance (11), and a fastidious nature (12), enterococcal infections became even more challenging to treat when these microbes are found in polymicrobial biofilm communities (13, 14). In addition, these polymicrobial biofilms can also serve as a nidus for systemic dissemination and a reservoir for the horizontal transfer of resistance genes (15). The robust nature of biofilms has posed major challenges in industrial, agricultural, and clinical settings (16). For these reasons, there has been a mounting interest in understanding the molecular processes driving species cooperation and competition within complex biofilm communities to inform tactics for clinical interventions (17).

While not exhaustive, previous studies have investigated microbial interactions of enterococci (13). In one study, it was reported that *Enterococcus faecalis*, the most prevalent enterococci in humans, suppresses *Clostridium perfringens* growth through the production of a bacteriocin (18). Around the same time, different groups showed that *E. faecalis* can inhibit the production of the botulinum neurotoxin by *Clostridium botulinum* (19), biofilm formation by *Streptococcus mutans* (20)*,* and virulence of the *Candida albicans* yeast in a biofilm-associated oropharyngeal candidiasis mouse model (21). Other studies have shown that co-culturing of *E. faecalis* with *Escherichia coli* results in suppression of pro-inflammatory responses (22), augmentation of the dual-species biofilm biomass (23), and heightened *E. coli* burden in a co-infection mouse incisional wound model (24). Another major opportunistic pathogen of infected wounds, *Pseudomonas aeruginosa* is frequently co-isolated with *E. faecalis* in polymicrobial wound infections (25). Like *E. coli*, co-culturing of *E. faecalis* and *P. aeruginosa* augmented biofilm biomass due to Psl- and Pel-mediated matrix polysaccharide production by *P. aeruginosa* (26). Paradoxically, a recent study indicated that *E. faecalis* inhibits *P. aeruginosa* growth by lowering environmental pH and L-lactate-mediated iron chelation (27). In an antibiotic-perturbed gastrointestinal mouse model, co-infection of *E. faecalis* with *C. difficile* enabled the sharing of fermentable amino acids, leucine and ornithine synthesized by *E. faecalis* while depletion of environmental arginine by *E. faecalis* acts as a metabolic cue to trigger upregulation of virulence in *C. difficile* (28). Finally, a recent study showed that exogenous heme released from the growth of *Staphylococcus aureus* augments *E. faecalis* biofilm, and this is facilitated by *E. faecalis* gelatinase activity that aids in the extraction of heme from *S. aureus* hemoproteins (29). Together, these studies reveal that specific bacterial pairs and the environmental context dictate whether *E. faecalis* interactions will be antagonistic or mutualistic.

Lactobacilli have also been shown to antagonize a variety of microbial pathogens (30–32). Due to their beneficial role in urogenital and gastrointestinal microbiome homeostasis, several members of this genus have been used as probiotics (33). Of note, lactobacilli (typically *L. crispatus, L. iners, L. gasseri*, or *L. jensenii*) (34, 35) constitute more than 50% of the microbial population of the vaginal tract, and their abundance is strongly associated with vaginal health (35, 36). In fact, depletion of lactobacilli in the vaginal microbiota is a clear sign of vaginal dysbiosis and, consequently, disease onset (37). The robust antagonistic capabilities of lactobacilli enable these commensal organisms to curb the outgrowth of potential vaginal pathogens and maintain vaginal eubiosis (38).

In recent years, *E. faecalis* has been implicated as an emerging vaginal pathogen (39, 40), with separate reports documenting its outgrowth and prevalence in vaginal swabs of patients suffering from bacterial vaginosis, and more recently, aerobic vaginitis (41, 42). Apart from a study implicating ethanolamine catabolism and the type VII secretion system in *E. faecalis* ability to colonize the vaginal tract (43), our understanding of the pathophysiology of *E. faecalis* in this microenvironment remains incomplete. Perturbation of vaginal microbiome homeostasis (44) and the depletion of the vaginal lactobacilli population (45) often precede the onset of vaginal infection. Concurring with this notion, a recent study by France and colleagues reported that vaginal microbial communities, sorted by community state types (CSTs), with overgrowth of vaginal pathogens (CST IV-C1, *Streptococcus* dominated; CST IV-C2, *Enterococcus* dominated; CST IV-C3, *Bifidobacterium* dominated; CST IV-C4, *Staphylococcus* dominated) have drastically reduced abundance of vaginal lactobacilli (35). Perhaps, by understanding the microbial interactions between *E. faecalis* and *L. crispatus*, a biomarker of vaginal health (46), we could then find explanations for *E. faecalis* ability to emerge as a pathogen in bacterial vaginosis. Using *in vitro* dual-species biofilm models, we show that *L. crispatus* eradicates *E. faecalis* temporally in a contact-independent manner. In addition, we showed that *E. faecalis-L. crispatus* co-infection protects the *Galleria mellonella* larvae against *E. faecalis*-mediated killing. Using transposon sequencing (Tn-seq) and RNA sequencing (RNA-seq) approaches, we identified specific genes and pathways that are either negatively or positively associated with the ability of *E. faecalis* to compete with *L. crispatus*. Altogether, this study represents a first step for the identification of adaptive traits that allows *E. faecalis* to mitigate assaults by lactobacilli, providing insights into some of the mechanisms that enable the persistence of *E. faecalis* in the vaginal tract.

## RESULTS

### *L. crispatus* eradicates *E. faecalis* in a temporal manner

**Fig 1:**
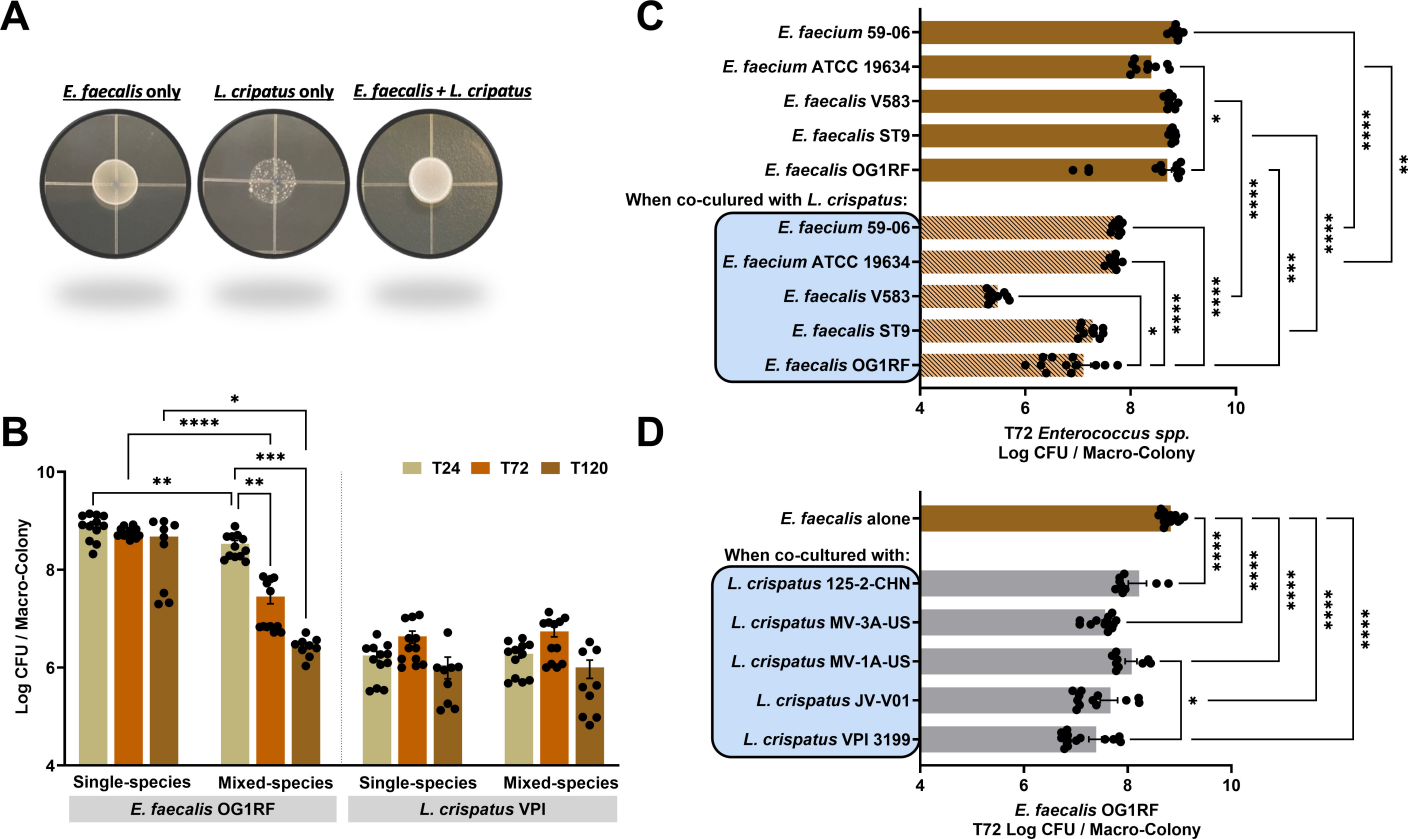
*L. crispatus* antagonizes the growth of enterococci. (**A**) Representative image of macro-colony biofilms on an MRS agar plate. (**B**) CFU recovered from *E. faecalis* OG1RF and *L. crispatus* VPI 3199 grown, respectively, either as single-species macro-colony biofilm or as dual-species (*E. faecalis + L. crispatus*) macro-colony biofilm for 24 h, 72 h, and 120 h post-incubation. (**C**) CFU recovered from selected *E. faecalis* and *E. faecium* strains, respectively, grown either as single-species macro-colony biofilm, or grown as dual-species macro-colony biofilms after 72 h incubation. (**D**) CFU recovered from *E. faecalis* OG1RF grown either as single-species macro-colony biofilm or grown respectively with selected *L. crispatus* strains as dual-species macro-colony biofilm after 72 h incubation. For B–D, data points represent 9–12 biological replicates, collated from at least three repeated experiments. Statistical analysis was performed using the Brown-Forsythe ANOVA test with Welch’s correction. Error bars represent standard error of the mean. *P ≤ 0.05, **P ≤ 0.01, *** *P* ≤ 0.001, **** *P* ≤ 0.0001.

Because the prevalence of *L. crispatus* is an indicator of a healthy vaginal tract (46), and *E. faecalis* is an understudied bacterial agent of vaginal infection (39, 40), we sought to investigate the microbial interactions between these two species. In the first series of experiments, we determined *E. faecalis* OG1RF and *L. crispatus* VPI 3199 growth and long-term viability as either single-species or mixed-species macro-colony biofilms (Fig. 1A). While *E. faecalis* alone grew to higher cell density as compared to *L. crispatus*, despite both species being inoculated at the same starting ratio, the viability of *L. crispatus,* alone or mixed with *E. faecalis* remained unchanged (Fig. 1B). However, there was a stepwise reduction in colony-forming units (CFUs) of *E. faecalis* when co-cultured with *L. crispatus* for 24 h (~0.5 log reduction), 72 h (~2 log reduction) and 120 h (~2.5 log reduction) that was not observed in the single-species *E. faecalis* macro-colony control (Fig. 1B). These results indicate that *L. crispatus* kills *E. faecalis* over time. To determine whether *L. crispatus-*mediated killing was conserved at the genus level, we probed the killing capacity of *L. crispatus* VPI 3199 against a panel of *E. faecalis* and *E. faecium* clinical and non-clinical strains. Like *E. faecalis* OG1RF, all other enterococcal strains lost viability when co-cultured with *L. crispatus,* with the vancomycin-resistant strain *E. faecalis* V583 showing the greatest rate of killing (~4 log after 72 h) (Fig. 1C). In addition, five different clinical strains of *L. crispatus* also showed equivalent capacities to kill *E. faecalis* OG1RF when mixed (Fig. 1D; Fig. S1). Lastly, we showed that other lactobacilli, *L. casei* and *L. rhamnosus*, can efficiently kill *E. faecalis* over time (Fig. S2). Besides macro-colony biofilms, we also grew *E. faecalis* OG1RF and *L. crispatus* VPI 3199 biofilms statically, either single or mixed species, using tissue culture plates for up to 72 h. In this experimental setup, *L. crispatus* VPI completely eradicated *E. faecalis* OG1RF after 72 h. Again, the viability of *L. crispatus* did not differ (Fig. S3). Finally, we also showed that *L. crispatus* can eradicate established (mature) *E. faecalis* biofilms (Fig. S4). Collectively, these results provide compelling evidence that *L. crispatus* and other lactobacilli can efficiently kill *E. faecalis* in dual-species biofilms *in vitro*.

### Mechanism of killing is contact-independent

**Fig 2:**
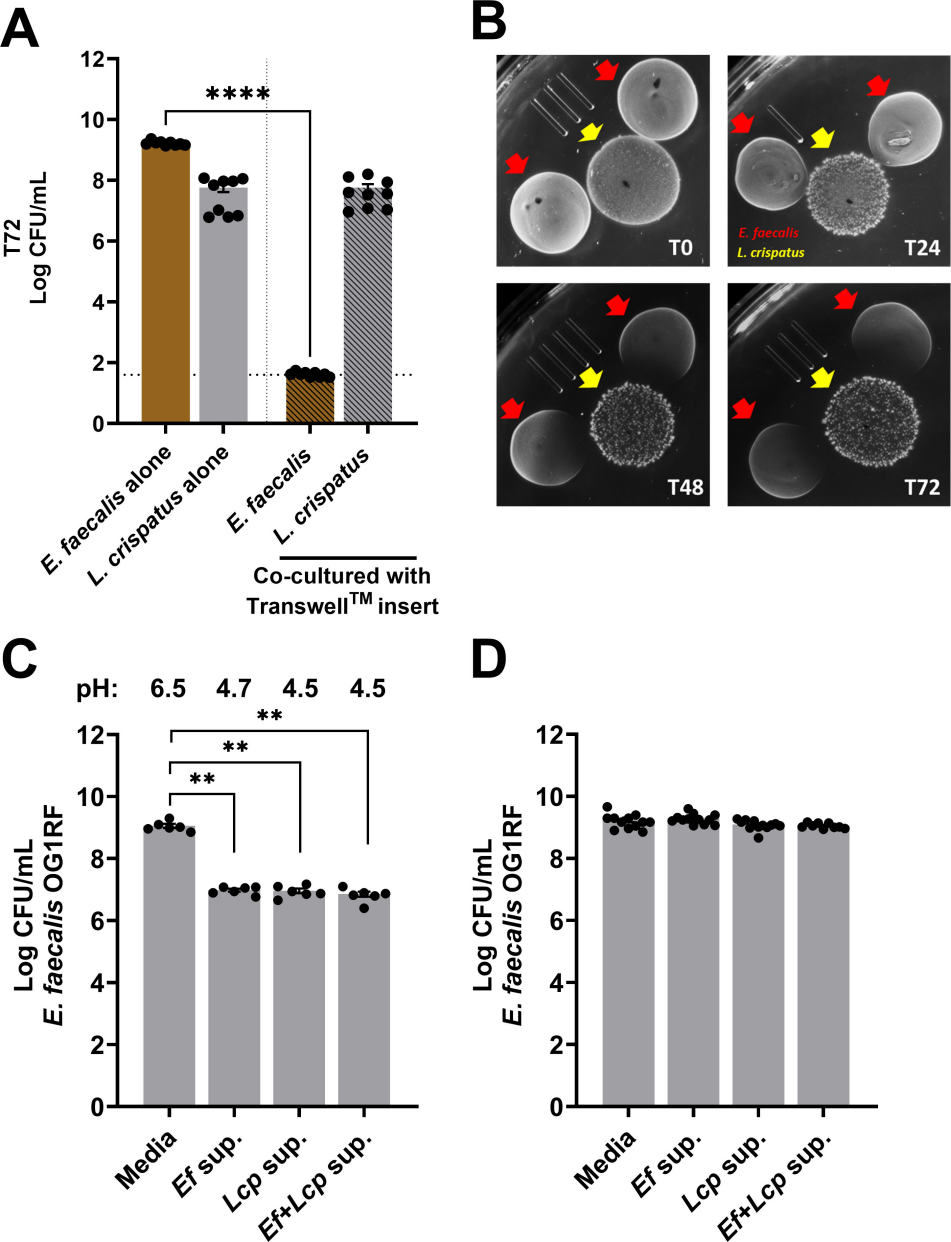
*L. crispatus* antagonism of *E. faecalis* is facilitated in a contact-independent manner. (**A**) CFU recovered from *E. faecalis* OG1RF and *L. crispatus* VPI 3199 grown statically for 72 h in MRS media as single-species biofilm, either in separate wells or in the same well separated by a Transwell membrane insert that prevents physical contact between cells. *L. crispatus* biofilm is grown on the flat surface of the well in the tissue culture plate, whereas *E. faecalis* biofilm is seeded on the surface of the Transwell membrane insert. The dotted line indicates the limit of detection (LOD), CFU < 42.5. (**B**) Representative images of spot antagonism assay showing growth inhibition of *E. faecalis* when *L. crispatus* macrocolony biofilms were established at the same time (T0), 24 h, 48 h, or 72 h before inoculating *E. faecalis*. CFU recovered from *E. faecalis* OG1RF growth after 24 h in MRS media mixed with 72 h cell-free biofilm supernatant isolated from single-species and dual-species biofilms at an equal ratio, either (**C**) pH-unadjusted or (**D**) adjusted to pH 6.5 to mirror the MRS media. For A, C, and D, data points represent 9–12 biological replicates, collated from at least three repeated experiments. Statistical analysis was performed using the Brown-Forsythe ANOVA test with Welch’s correction. Error bars represent the standard error of the mean. **P ≤ 0.01, ****P ≤ 0.0001.

To obtain insights into the mechanism(s) of *L. crispatus*-mediated killing of *E. faecalis*, we performed a series of *in vitro* static biofilm experiments using the 72 h time point (~7 log killing, refer to Fig. S3) as the read-out. To probe whether killing is contact-dependent, we grew single-species *E. faecalis* and *L. crispatus* biofilms together in a tissue culture plate, separated by a Transwell permeable membrane insert that prevents physical contact while allowing the exchange of metabolites and other types of molecules in the same well. Because CFUs recovered from *E. faecalis* biofilms seeded on the surface of the Transwell membrane insert were drastically reduced (~7 log; similar levels to those observed in Fig. S3), while CFUs of *L. crispatus* biofilms at the bottom of the culture plate remained unchanged (Fig. 2A), we concluded that secreted molecule(s) are responsible for the eradication of *E. faecalis*. Next, we switched to a semi-solid agar plate-based macro-colony biofilm antagonism assay to investigate if *L. crispatus* secreted, diffusible molecule(s) are produced in the absence of *E. faecalis*. While simultaneous inoculation of *E. faecalis* and *L. crispatus* did not result in a zone of clearing at T0 and minimally affected *E. faecalis* growth when inoculated 24 h later (T24), spotting of *E. faecalis* macro-colony biofilms 48 and 72 h after the growth of *L. crispatus* macro-colony biofilms resulted in clear zones of inhibition (Fig. 2B). This result indicates that *L. crispatus*-mediated growth inhibition of *E. faecalis* occurs at later time points and is not associated with the presence of *E. faecalis*. While *L. crispatus* bacteriocins have not been described, previous studies have identified phenyl-lactic acid (PLA), present in cell-free supernatants of *L. crispatus*, as a bactericidal compound with wide spectrum activity against other bacteria, including *E. faecalis* (47). PLA was shown to cause cell membrane damage providing, at least in part, an explanation for its antimicrobial activity (48). To test whether PLA produced by lactobacilli can be accounted for the killing of *E. faecalis* observed here*,* we determined the inhibitory concentration of PLA against *E. faecalis* OG1RF or *L. crispatus* VPI. Based on the observation that similar concentrations of PLA were inhibitory to both species (Fig. S5), we conclude that it is unlikely that *L. crispatus* secreted PLA alone is responsible for the killing of *E. faecalis*. Moreover, enterococci also secrete PLA, and the extracellular concentrations of PLA produced by either lactobacilli or enterococci that have been reported previously (49, 50), approximately 1 mM (0.16 mg/mL), is not inhibitory to *E. faecalis*.

**Fig 3:**
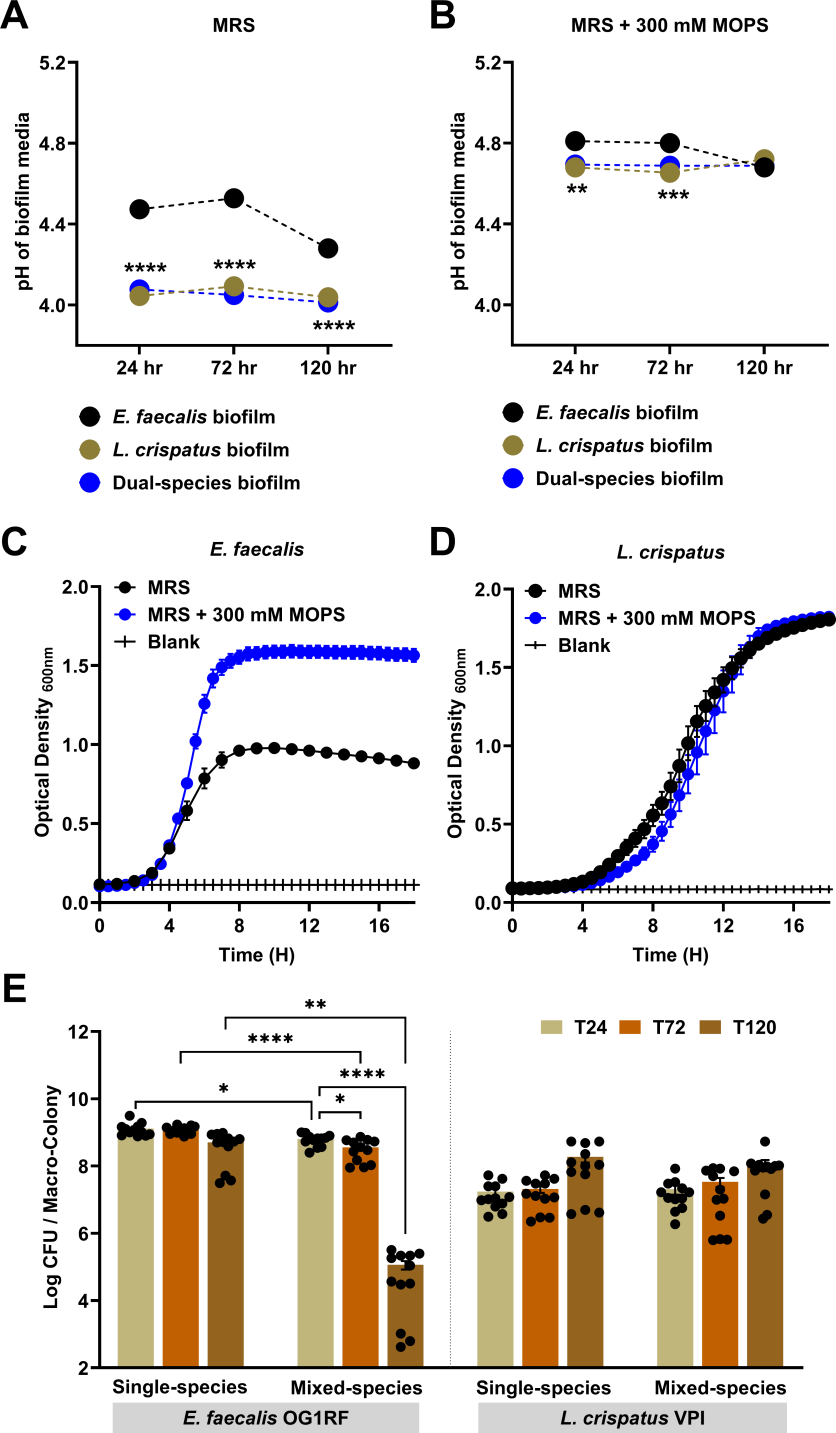
*L. crispatus* antagonistic activity is enhanced in an aciduric environment. pH measurements from *E. faecalis* OG1RF and *L. crispatus* VPI 3199 grown statically between 24 and 120 h either as single-species biofilm or as dual-species (*E. faecalis + L. crispatus*) biofilm in MRS (**A**) or MRS supplemented with 300 mM MOPS buffer (**B**). Corresponding growth dynamics of *E. faecalis* (**C**) and *L. crispatus* (**D**) in MRS or MRS supplemented with 300 mM MOPS buffer. (**E**) CFU recovered from *E. faecalis* OG1RF and *L. crispatus* VPI 3199 grown, respectively, either as single-species macro-colony biofilm or as dual-species (*E. faecalis + L. crispatus*) macro-colony biofilm for 24 h, 72 h, and 120 h post-incubation in MRS agar supplemented with 300 mM MOPS. For A–D, data points represent 9–12 biological replicates, collated from at least three repeated experiments. For A and B, statistical analysis was performed using two-way ANOVA. For C and D, linear regression of the slope of the exponential growth phase was performed. For E, statistical analysis was performed using the Brown-Forsythe ANOVA test with Welch’s correction. Error bars represent the standard error of the mean. *P ≤ 0.05, **P ≤ 0.01, *** *P* ≤ 0.001, **** *P* ≤ 0.0001.

Because *E. faecalis* viability is drastically reduced after 72 h post-incubation in co-culture (Fig. 2A), we reasoned that the *L. crispatus* anti-enterococcal diffusible molecule(s) might be of most abundant at this time point. To test this possibility, we mixed cell-free biofilm supernatants collected from either single or mixed species *L. crispatus* biofilms grown for 72 h, with fresh MRS in a 50:50 ratio to monitor *E. faecalis* growth and viability. Because of the possibility that the acidic pH of the biofilm supernatants may contribute to the killing activity of *L. crispatus* anti-enterococcal diffusible molecule(s), we left the pH unchanged (*Ef* sup, pH 4.7; *Lcp* sup and *Ef +Lcp* sup, pH 4.5; refer to Fig. 3A). While we were surprised that the CFUs recovered from the growth of *E. faecalis* in *L. crispatus* (*Lcp* sup) or mixed-species (*Ef +Lcp* sup) supernatant were similar to CFUs recovered from growth in its biofilm supernatant, it is not startling that the acidic pH of these cell-free biofilm supernatants impeded the overall growth of *E. faecalis* when compared to the media control (Fig. 2C). To rule out that the growth impairment due to acidic pH undermined, our ability to observe reduction in enterococcal CFUs due to *L. crispatus* anti-enterococcal diffusible molecule(s), we adjusted the pH of the biofilm supernatants to match those of the MRS media (pH 6.5). While the overall growth of *E. faecalis* is no longer impeded, compared to its pH-adjusted biofilm supernatant (*Ef* sup) and media control, the viability of *E. faecalis* when grown in *L. crispatus* (*Lcp* sup) or mixed-species (*Ef +Lcp* sup) pH-buffered supernatant, again did not differ (Fig. 2D). We then increased the biofilm supernatant to media ratio (90:10); still, we did not observe any reduction in CFUs of *E. faecalis* (Fig. S6). Altogether, we show that the mechanism of killing is contact-independent. However, under these conditions tested, we are unable to verify the presence of *L. crispatus* secreted anti-enterococcal diffusible molecule(s) in biofilm supernatants.

### Acidic environment enhances antagonistic activity but is not accountable for the killing of *E. faecalis*

Because lactic acid production by lactobacilli and concomitant environmental acidification has been shown to inhibit the growth of other bacteria and is associated with vaginal eubiosis (38, 51), we next asked whether the lower pH environment created by *L. crispatus* is responsible for the killing of *E. faecalis*. First, we measured the pH of *E. faecalis*, *L. crispatus,* and *E. faecalis-L. crispatus* biofilm supernatants at 24, 72, and 120 h. As expected, *L. crispatus* alone or in co-culture with *E. faecalis* lowered the culture pH (~4.2) to values below those achieved by *E. faecalis* alone (~4.3) by 120 h post-incubation (Fig. 3A). We do not anticipate that acidic pH plays a role in the eradication of *E. faecalis* because of two reasons; first, killing of *E. faecalis* occurs temporally (Fig. 1B; Fig. S3), and second, acidic pH (in static biofilms) remained relatively similar throughout the duration of the experiment (Fig. 3A), as is expected of these aciduric bacteria, whether alone or mixed. Nonetheless, to rule out that the lower final pH of ~4.0 driven by *L. crispatus* is implicated in the killing of *E. faecalis* in the mixed-species biofilm, we used 300 mM MOPS (3-(N-morpholino) propanesulfonic acid) to buffer the biofilm pH to around ~4.6 (*Ef* alone) and ~4.8 (*Lcp* alone or in co-culture), with all culture conditions reaching the same pH after 120 h (Fig. 3B). While the addition of MOPS enhanced the growth of *E. faecalis in vitro,* there was no discernible effect on *L. crispatus* growth (Fig. 3C and D). As we anticipated, the addition of MOPS to MRS agar plates did not rescue *E. faecalis* from eradication in mixed-species macro-colony biofilms nor did it affect the viability of *L. crispatus*, albeit reduction in CFUs of *E. faecalis* in co-culture at 72 h was less drastic (0.5-log; Fig. 3E; compared to 2-log reduction in MRS agar, Fig. 1B). Furthermore, the addition of MOPS had no impact on *L. crispatus* ability to eradicate established *E. faecalis* biofilms (Fig. S4). Altogether, these results indicate that an acidic environment accelerates but is not the main reason for the killing of *E. faecalis* by *L. crispatus*.

### Identification of transcriptional patterns that enable *E. faecalis* to compete with *L. crispatus*

**Fig 4:**
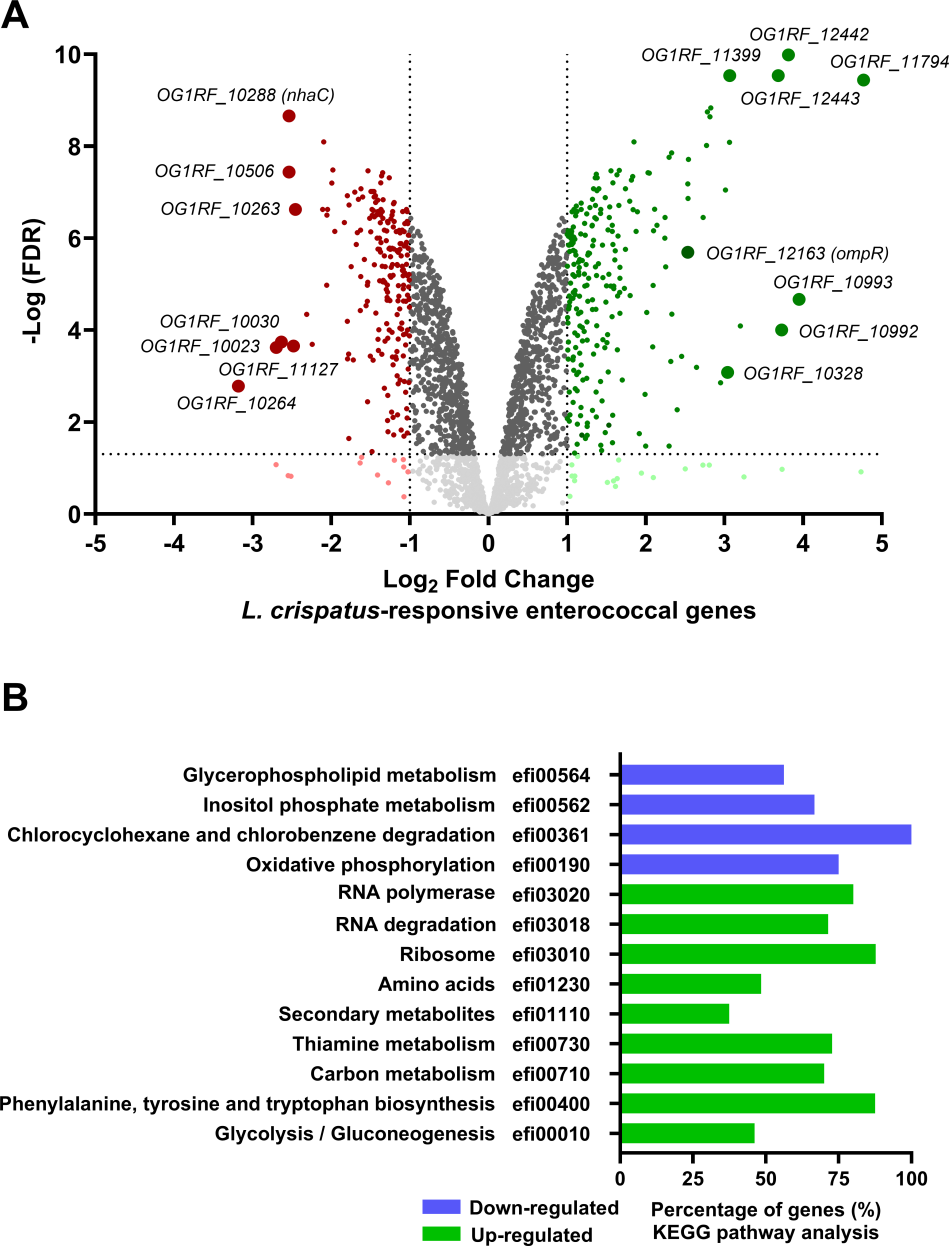
Global transcriptomic changes of *E. faecalis* when co-cultured with *L. crispatus*. (**A**) Graphical representation of the total number of genes transcriptionally changed relative to the degree of log_2_ fold change observed. (**B**) KEGG pathway analysis of the most significant changes in metabolic pathways. Data shown are obtained from testing three biological replicates. The total number of genes in each KEGG pathway is listed in Table S2. Refer to methods for information on analysis.

To obtain insights into how *E. faecalis* responds to *L. crispatus* antagonism, we used RNA sequencing (RNA-seq) to identify transcriptional networks of *E. faecalis* that are altered in *L. crispatus-E. faecalis* dual-species macro-colony biofilms. By comparing gene expression profiles of *E. faecalis* grown singly or mixed with *L. crispatus* for 24 h, with a cut-off of log_2_FC of ±1 and false discovery rate (FDR) of 0.05, we identified 201 genes downregulated and 218 genes upregulated (Fig. 4A; Table S1). The most upregulated and downregulated genes, which comprise the top 50 most differentially expressed genes (Table 1), according to KEGG pathway analysis (Fig. 4B; Table S2; *P*-value ≤0.05), were found to be involved in glycerophospholipid metabolism, carbon and sugar utilization, central metabolism, and general stress responses. Among the 20 highly upregulated genes were the response regulator *ompR* (*OG1RF_12163*), two hypothetical proteins (*OG1RF_11794, OG1RF_11399*), putative LysM domain-containing peptidoglycan hydrolase (*OG1RF_10328*), FMN reductase (*OG1RF_12442*), Rrf2 family transcriptional regulator (*OG1RF_12443*), and spermidine/putrescine ABC family transporter (*OG1RF_10991–10993*), while the remaining genes encode for proteins involved in central metabolism. Among the 20 most downregulated genes were malate quinone oxidoreductase (*OG1RF_10264*), cytochrome bd-l oxidoreductase (*OG1RF_11665*), four hypothetical proteins (*OG1RF_11127, OG1RF_10506, OG1RF_10023,* and *OG1RF_10263*), an alanine tRNA ligase (*OG1RF_t10030*), and a putative Na^+^:H^+^ antiporter (NhaC; *OG1RF_10288*), while the remaining genes encode for proteins involved in sugar and carbon transport (Fig. 4A).

**TABLE 1:** Select list of the most differentially expressed genes during co-culture with *L. crispatus* relative to *E. faecalis* single-species control

Old locus tag	Gene ID	Description	Log_2_FC	*P*-value	FDR
Sugar and carbon utilization/uptake					
OG1RF_10030		ABC superfamily ATP binding cassette transporter permease subunit	−2.6351	5.32E-05	1.82E-04
OG1RF_10684		Maltose PTS enzyme II	2.2417	6.60E-08	1.00E-06
OG1RF_10991		Spermidine/putrescine ABC transporter permease protein	3.2017	2.08E-05	8.18E-05
OG1RF_10992		Spermidine/putrescine ABC transporter ATP-binding protein	3.7275	2.59E-05	9.94E-05
OG1RF_10993		Spermidine/putrescine ABC transporter substrate-binding protein	3.9476	3.96E-06	2.14E-05
OG1RF_10994	ade	Adenine deaminase	2.4560	1.26E-04	3.75E-04
OG1RF_11022	trpX	Tryptophan ABC transporter substrate-binding protein	2.8249	3.55E-12	1.48E-09
OG1RF_11023		Tryptophan ABC transporter permease protein	2.2970	1.05E-10	1.75E-08
OG1RF_11024		Tryptophan ABC transporter ATP-binding protein	3.0645	3.95E-11	8.23E-09
OG1RF_11133	mdxG	Sugar ABC transporter permease	2.3167	1.72E-04	4.84E-04
OG1RF_11134	mdxF	Sugar ABC transporter permease	2.3274	7.82E-11	1.40E-08
OG1RF_11135	mdxE	Extracellular maltose-binding protein	3.0676	4.60E-13	2.92E-10
OG1RF_11136		Glycoside hydrolase family 13 protein	2.5327	9.58E-10	6.66E-08
OG1RF_11137		Alpha-glucosidase	2.2435	1.44E-08	3.56E-07
OG1RF_11234		PTS enzyme IIB component	2.9516	5.79E-04	1.40E-03
OG1RF_11542		Putative group I ECF transporter transmembrane (T) component	−2.0465	7.37E-09	2.38E-07
OG1RF_11598		PTS enzyme IIBC component	2.5367	2.92E-09	1.38E-07
OG1RF_11753	treP	Putative trehalose-specific PTS enzyme EIIBC component	2.7823	5.03E-12	1.80E-09
OG1RF_11763		ABC transporter permease	−2.2422	6.13E-05	2.05E-04
OG1RF_12087		D-aminopeptidase superfamily protein	2.2514	4.84E-07	4.23E-06
OG1RF_12088		Putative glutamyl aminopeptidase	2.7273	1.42E-08	3.55E-07
OG1RF_12089		Oligopeptide ABC superfamily ATP-binding cassette transporter, binding protein	3.0122	1.47E-09	9.00E-08
Transcriptional regulators					
OG1RF_12163	ompR	OmpR family response regulator transcription factor	2.5336	1.78E-07	2.04E-06
OG1RF_12443		Rrf2 family transcriptional regulator	3.6850	3.19E-13	2.92E-10
OG1RF_12573	citO	DNA-binding transcriptional regulator	−2.0584	1.58E-06	1.06E-05
Glycolipid modification					
OG1RF_10094	sdaA	L-serine ammonia-lyase, alpha subunit	2.1115	6.47E-09	2.35E-07
OG1RF_10095	serS	Serine tRNA ligase	2.0975	3.72E-08	6.91E-07
OG1RF_10030		tRNA-Ala	−2.6351	5.32E-05	1.82E-04
OG1RF_10328		LysM domain-containing protein	3.0385	3.24E-04	8.38E-04
OG1RF_12461	lrgA	Antiholin-like murein hydrolase modulator	2.3980	2.64E-03	5.43E-03
OG1RF_12506		LPXTG cell wall anchor domain-containing protein	2.8139	8.31E-12	2.31E-09
Metabolism and stress response					
OG1RF_10288	nhaC	Putative transporter, Na+:H + antiporter family	−2.5366	7.05E-12	2.21E-09
OG1RF_10597	gloA4	Lactoylglutathione lyase	2.1406	2.57E-08	5.26E-07
OG1RF_10837		AAA family ATPase	−2.1085	7.13E-09	2.38E-07
OG1RF_11349	pduQ	1-propanol dehydrogenase PduQ	2.6438	2.42E-04	6.50E-04
OG1RF_11665	cydB	Cytochrome bd-I demethylquinol:oxygen oxidoreductase subunit II	−2.0955	3.47E-11	8.12E-09
OG1RF_12165	aadK	Aminoglycoside 6-adenylyltransferase	2.5423	1.25E-10	1.95E-08
OG1RF_12442		NAD(P)-dependent oxidoreductase	3.8137	4.14E-14	1.04E-10
OG1RF_12545	rsmG	16S rRNA (guanine(527)-N(7))-methyltransferase RsmG	2.0277	3.49E-10	3.80E-08
Unknown function					
OG1RF_10023		DUF308 domain-containing protein	−2.4806	6.75E-05	2.23E-04
OG1RF_10264		DUF4822 domain-containing protein	−2.4556	7.27E-09	2.38E-07
OG1RF_10506		Hypothetical protein	−2.5353	3.08E-10	3.67E-08
OG1RF_10950		Hypothetical protein	2.0118	9.19E-06	4.16E-05
OG1RF_11127		Hypothetical protein	−2.6977	7.43E-05	2.41E-04
OG1RF_11263		Helix-turn-helix domain-containing protein	2.0430	3.69E-10	3.85E-08
OG1RF_11399		Hypothetical protein	3.0745	4.67E-13	2.92E-10
OG1RF_11794		DoxX family protein	4.7674	7.27E-13	3.64E-10
OG1RF_12414		LXG toxin	2.2947	2.01E-02	3.33E-02
OG1RF_12455		TIGR00282 family metallophosphoesterase	2.0867	7.09E-05	2.32E-04
OG1RF_11937		Hypothetical protein	2.7725	5.05E-11	9.73E-09

### Tn-seq identifies genes that enable *E. faecalis* to compete with *L. crispatus*

Next, we used Tn-seq (52) to identify *E. faecalis* OG1RF transposon mutants that are either under-represented (sensitivity to killing) or over-represented (resistant to killing) when co-cultured with *L. crispatus*. Using an FDR < 0.05 and Monte Carlos value of 0.373, eight mutants were under-represented at 24 h. Remarkably, all mutants contained insertions within the *dltA*, *dltB,* or *dltD* genes (Table S3). The *dltABCD* operon (*OG1RF_12109–12112*) is responsible for the D-alanylation of cell wall teichoic acid, a modification that is important for virulence and antimicrobial peptide and daptomycin tolerance in Gram-positive bacteria (53). Using the same cutoff applied to the 24 h time point, 30 Tn mutants were identified at 48 h, with three Tn mutants under-represented (*OG1RF_10193, OG1RF_10681,* and *hexB*) and the remaining Tn mutants over-represented (Table S4). Among the over-represented genes with multiple hits, five had insertions within *ldh1* (*OG1RF_10199*), coding for the lactate dehydrogenase, two within *ndh2* (*OG1RF_11660*) coding for NADH dehydrogenase, and 12 within the *dltABCD* operon. At 72 h post-incubation, 6 Tn mutants identified were under-represented, with 2 hits in genes of unknown function (*OG1RF_10435* and *OG1RF_11674*) and the remaining with disruptions in genes that contribute to different metabolic processes (Table S5).

**Fig 5:**
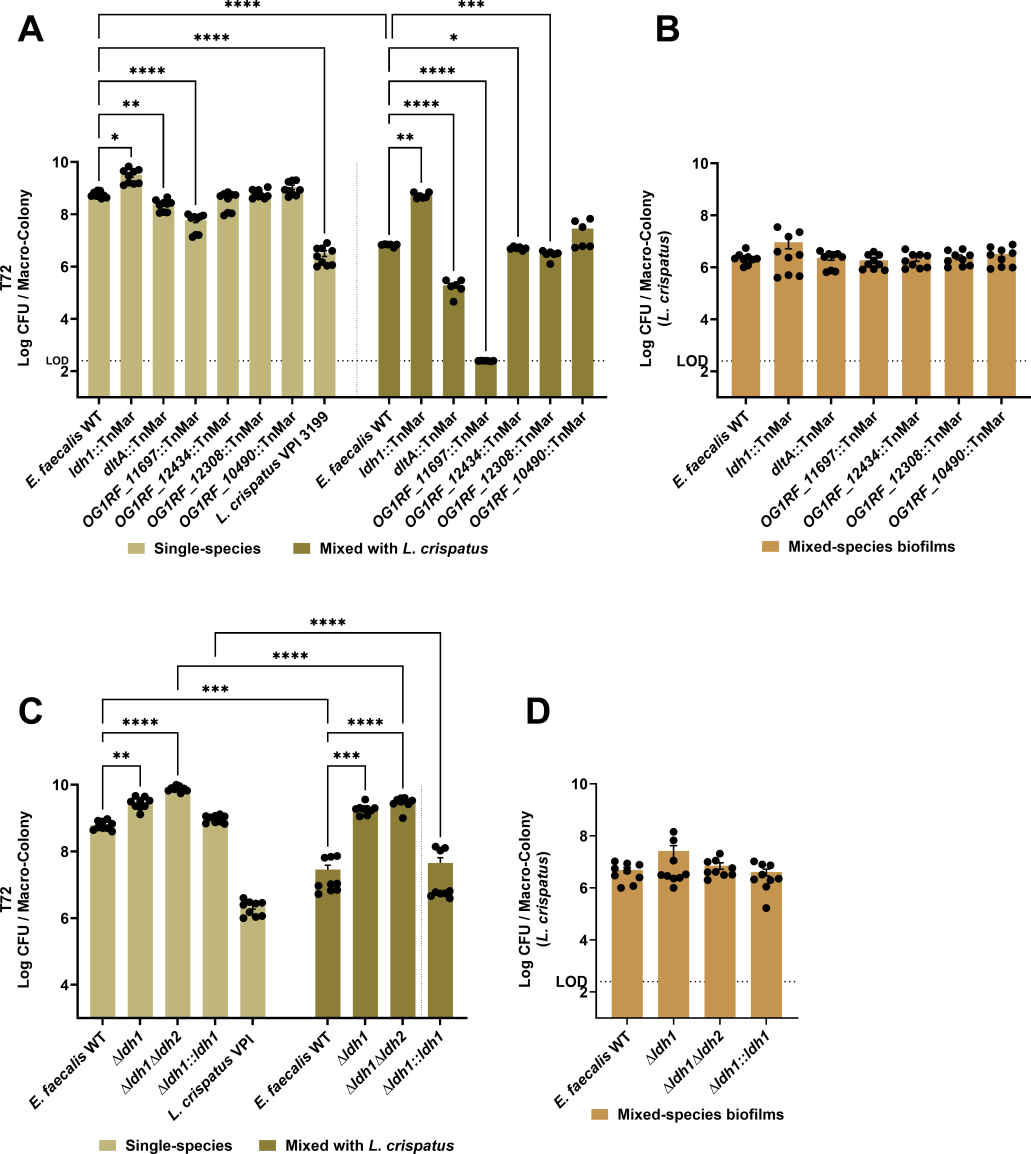
*E. faecalis* genes contribute to tolerance against *L. crispatus* antagonism. CFU recovered from *E. faecalis* OG1RF, its isogenic transposon mutants, and *L. crispatus* VPI 3199 grown, respectively, either as single-species macro-colony biofilm or as dual-species (*E. faecalis +L. crispatus*) macro-colony biofilm (**A and B**) for 72 h. CFU recovered from *E. faecalis* OG1RF, its isogenic LDH deletion, and complementation mutants, as well as *L. crispatus* VPI 3199 grown respectively either as single-species macro-colony biofilm or as dual-species (*E. faecalis +L. crispatus*) macro-colony biofilm (**C and D**) for 72 h. Data points represent 9–12 biological replicates, collated from at least three repeated experiments. Statistical analysis was performed using the Brown-Forsythe ANOVA test with Welch’s correction. Error bars represent the standard error of the mean. *P ≤ 0.05, **P ≤ 0.01, *** *P* ≤ 0.001, **** *P* ≤ 0.0001.

Because insertions within the *dltABCD* operon and *ldh1* represented the biggest pool of Tn mutants identified, we accessed the *E. faecalis* arrayed transposon library (54) to retrieve individual *dltA* and *ldh1* Tn mutants in addition to three other Tn mutants (OG1RF_11697, OG1RF_10490, and *hexB*) that appeared at the 48 h and 72 h time point (Table 2) for validation purposes. By individually co-culturing these Tn mutants with *L. crispatus*, we verified that the *dltA::*TnMar was indeed more susceptible to *L. crispatus* killing, showing ~1 log reduction in CFU recovered after 72 h when compared to the survival of the parent strain (Fig. 5A). In agreement with the TnSeq hits, the *OG1RF_11697*::TnMar and *ldh1*::TnMar were, respectively, were more sensitive (6 log) and more resistant (~1 log) to *L. crispatus* killing. While statistically significant, Tn mutants with disruption in *OG1RF_12434* and *OG1RF_12308* were not drastically susceptible (~1 fold reduction) to *L. crispatus*-mediated killing (Fig. 5A). Again, 72 h viability of *L. crispatus* remained unchanged when co-cultured with any of these mutants (Fig. 5B).

**TABLE 2:** Selected list of differentially abundant *E. faecalis* transposon mutants during co-culture with *L. crispatus* relative to *E. faecalis* single-species counterpart

Old locus tag	Gene name	NCBI description	Single vs mixed t24 (Log_2_FC)	Single vs mixed t48 (Log_2_FC)	Single vs mixed t72 (Log_2_FC)
OG1RF_12109	dltD	D-alanyl-lipoteichoic acid biosynthesis protein	−1.21	1.72	ns
OG1rf_12111	dltB	D-alanine transfer protein	−1.60	1.76	ns
OG1RF_12112	dltA	D-alanine--D-alanyl carrier protein ligase	−1.46	1.29	ns
OG1RF_10199	ldh1	L-lactate dehydrogenase	ns	2.42	ns
OG1RF_11697		Hypothetical protein	ns	5.28	ns
OG1RF_12434	hexB	DNA mismatch repair protein	ns	−1.26	ns
OG1RF_12435		Hypothetical protein	ns	ns	−1.38
OG1RF_12308		Thioredoxin family protein	ns	ns	−4.21
OG1RF_12309	pepA2	Glutamyl aminopeptidase	ns	ns	−0.94
OG1RF_10490		Cell wall surface anchor family protein	ns	3.06	−4.21

In previous studies, *E. faecalis* LDH (known as Ldh1) was found to be important for iron-augmented energy production (55) and for the inhibition of *P. aeruginosa* growth via L-lactate-dependent iron chelation (27). In addition, a second LDH gene (*OG1RF_10373, ldh2*) is present in the OG1RF genome, sharing 46% similarity with Ldh1 (27). Even though Ldh1 has been found to play a prominent role in lactate production, both Ldh1 and Ldh2 have redundant activities participating in L-lactate production and redox maintenance (27, 56, 57). As further validation of the Tn screen, we tested the capacity of in-frame deletion strains lacking one or both *ldh* genes (∆*ldh1*, *∆ldh1∆ldh2*) as well as a genetically complemented *∆ldh1* strain (*∆ldh1::ldh1*) (27) to compete with *L. crispatus* using the macro-colony biofilm plate assay. As previously reported (27), inactivation of *ldh1* alone or in combination with *ldh2* enabled *E. faecalis* to grow to higher cell density (Fig. 5C). Consistent with the TnSeq validation results, loss of *ldh1* alone, or both *ldh1* and *ldh2*, restored *E. faecalis* viability in the *L. crispatus-E. faecalis* macro-colony assay to levels observed in the single *E. faecalis* macro-colonies. Furthermore, genetic complementation of *ldh1* (*∆ldh1::ldh1*) restored sensitivity to killing as compared to the survival rate of the parent strain (Fig. 5C). Again, 72 h viability of *L. crispatus* remained unchanged when co-cultured with any of these mutants (Fig. 5D).

**Fig 6:**
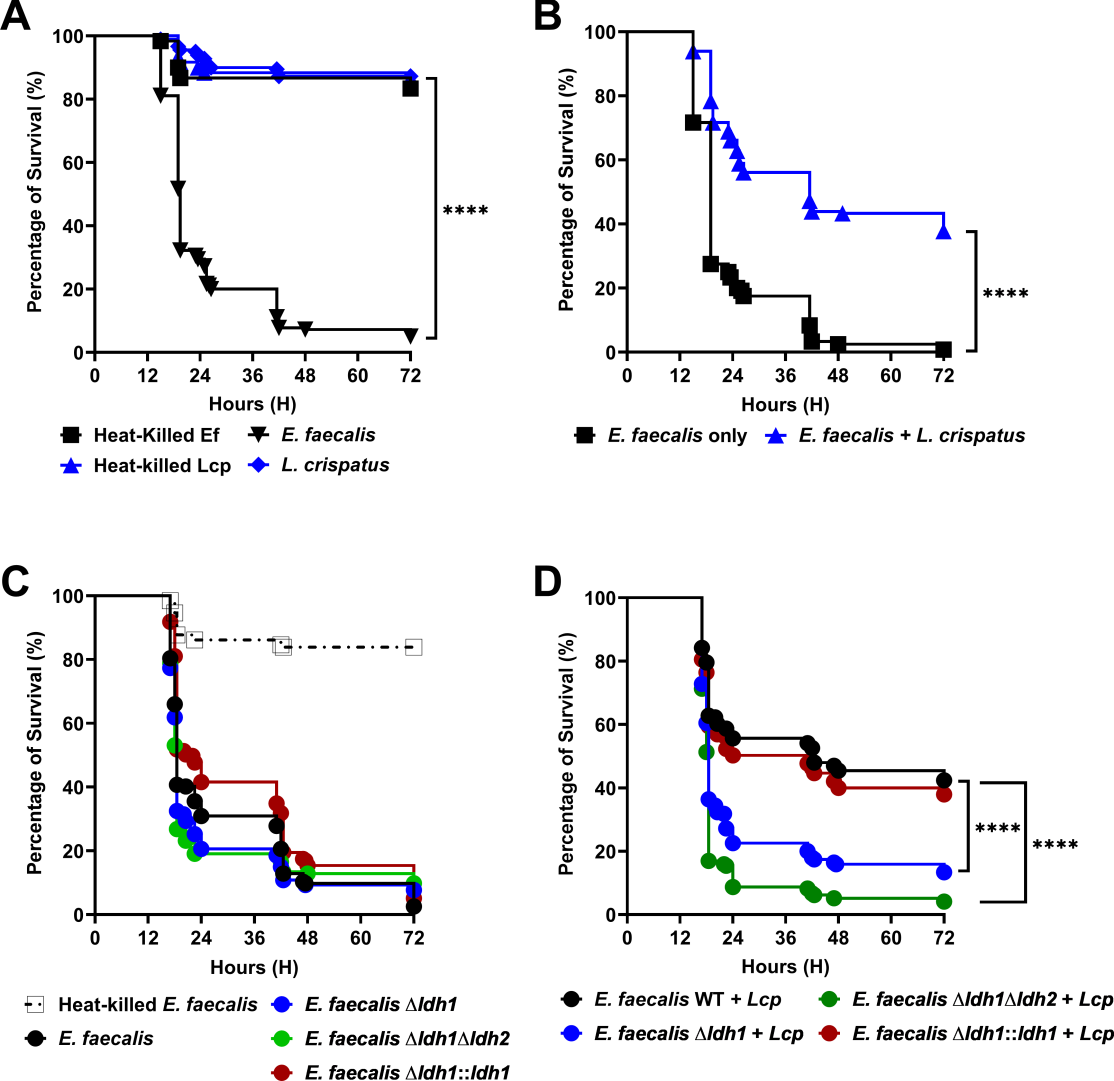
Loss of *ldh1* restores virulence of *E. faecalis* in a co-infection model of Galleria larvae. Percentage survival of *G. mellonella* larvae 72 h post-injection with either single-species controls; *E. faecalis* OG1RF or *L. crispatus,* and their heat-killed counterparts (**A**), or co-injected with both (**B**). Percentage survival of *G. mellonella* larvae 72 h post-injection with *E. faecalis* OG1RF parent strain and its isogenic LDH deletion and complementation strains alone (**C**), or their corresponding co-injected counterparts (**D**). In each *Galleria* infection experiment, 20 larvae were infected with one biological replicate of *E. faecalis* inoculant, with a total of three replicates per experiment. Data points represent 9 biological replicates of *E. faecalis* inoculum, repeated thrice (*n* = 3) on non-consecutive days. Each curve represents a group of 180 larvae, individually injected with 10^5^ CFUs of *E. faecalis* suspended in PBS at a final volume of 5 µL. Statistical analysis was performed using the log-rank (Mantel-Cox) test. ****P ≤ 0.0001.

Because the prevalence of lactobacilli is linked to urogenital and gastrointestinal microbiome homeostasis (30–32, 58), and these microbes have been used as probiotics (30–33, 58–62), in the next set of experiments, we explored the usefulness of the *Galleria mellonella* larvae model to examine the potential of *L. crispatus* to protect the *Galleria* larvae against *E. faecalis* lethal infection. First, we showed that *L. crispatus* does not kill *G. mellonella* as larvae inoculated with *L. crispatus* alone displayed similar survival rates as larvae injected with heat-killed bacteria (Fig. 6A). Remarkably, co-inoculation of *L. crispatus* with *E. faecalis* enhanced larvae survival by ~40% when compared to larvae inoculated with an identical dose of *E. faecalis* alone (Fig. 6B). Next, we tested the virulence potential of the Δ*ldh1,* Δ*ldh2,* and Δ*ldh1*Δ*ldh2* strains alone or co-inoculated with *L. crispatus*. While not statistically significant, all *ldh* mutants killed *G. mellonella* faster than parent or Δ*ldh1::ldh1* strains (Fig. 6C). More importantly, the protection conferred by *L. crispatus* was completely lost in larvae co-infected with *L. crispatus* and any one of the *ldh* mutants (Fig. 6D).

## DISCUSSION

Fundamental knowledge of microbial interactions is essential and has strong implications for the design of interventions for biofilm-associated infections (13, 63). While *L. crispatus* is a biomarker of vaginal health, *E. faecalis* has been implicated as a vaginal pathogen in recent years. Though it is likely that *E. faecalis* takes advantage of a vaginal biome with lower counts of lactobacilli to enable colonization of the vaginal tract and, ultimately, drive the onset of aerobic vaginitis (39, 40, 64), the molecular mechanisms as well as the pathophysiology of *E. faecalis* in the vaginal tract remain unknown. By leveraging high-throughput molecular approaches to identify genetic factors that contribute to competitive interactions between *E. faecalis* and *L. crispatus*, we will gain a better understanding of how *E. faecalis* mitigate assaults by *L. crispatus*.

Several studies have shown that cell-free supernatants of *L. crispatus* inhibit the growth of vaginal pathogens, including *Gardnerella vaginalis* (65), *Candida albicans* (66), and *Streptococcus agalactiae* (67, 68). In *S. agalactiae,* a major pathogen of vaginal infections, this inhibitory effect was largely attributed to the lower tolerance of *S. agalactiae* to the more acidic pH environment generated by the presence of lactobacilli (68). Though our results did not completely concur with findings from other groups that cell-free supernatants indeed have growth-inhibitory properties, it is certain that *L. crispatus* uses a contact-independent mechanism to kill *E. faecalis*. Perhaps, the molecule(s) responsible for killing *E. faecalis* might be labile; or alternatively, at inadequate doses in the supernatants. It is also plausible that acidic pH, PLA (47), and other complementary mechanisms that we are unaware of, work in a concerted effort with these unknown molecule(s) to kill *E. faecalis*. Besides using antimicrobial molecules to curb the outgrowth of vaginal pathogens, one recent study also noted that co-culturing with *L. crispatus* reduces *S. agalactiae* recto-vaginal colonization and attenuate invasion of endometrium decidual cells (69), though mechanisms remained to be determined, providing alternative explanations for *L. crispatus* ability to dominate the vaginal tract. In a similar context, a recent report also revealed that *L. crispatus* actively outcompetes *E. faecalis* to adhere to vaginal epithelial cells (70). While studies of *L. crispatus* are limited, *L. rhamnosus* which primarily cohabits the gastrointestinal tract has been extensively studied for its antagonistic capabilities (62) against other resident members of the gastrointestinal tract. Of these studies, the antagonistic mechanisms are speculated to be linked to the secretion of bacteriocins, organic acids, or hydrogen peroxide, which are traits shared among members of the genus *Lactobacillus* (38, 51, 62). Concurring with previous studies that probe the potential of lactobacilli as probiotics (33, 59–61), we also demonstrated that co-injection of *L. crispatus* with *E. faecalis* significantly enhanced *G. mellonella* larvae survival, and this *L. crispatus-*mediated protection was lost when larvae were co-injected with *L. crispatus* and isogenic *E. faecalis* LDH mutants that were resistant to *L. crispatus*-mediated killing. While results are promising, whether phenotypes will be recapitulated in a vaginal infection model remains to be addressed and will be pursued in the future. Though this report focuses on how *E. faecalis* perceives the presence and attempt to compete with *L. crispatus*, studies to identify the molecular factors responsible for the killing of *E. faecalis* by *L. crispatus* will soon be underway.

By leveraging the killing ability of *L. crispatus* as a tool for the Tn-seq screen*,* we identified *E. faecalis* genes that are important for competition against *L. crispatus*. We found that *E. faecalis* Tn mutants with disruption in the *dltABCD* operon or *OG1RF_11697* (hypothetical protein) were more susceptible to *L. crispatus* killing, whereas Tn mutants with disruption in the *ldh1* gene showed increased resistance. The *dltABCD* gene products that catalyze the addition of d-alanine esters to wall teichoic acids (71) and lipoteichoic acids (LTA) (72), two important components of the cell wall of Gram-positive bacteria, also play vital roles in a number of biological processes, such as autolysis and biofilm formation (72). Most importantly, the D-alanylation of teichoic acids provides resistance to cationic antimicrobial peptides (CAMP), by raising the overall net negative charge of the membrane, thereby reducing the affinity for CAMP (73). Since the loss of *dltA* in *E. faecalis,* which encodes for a D-alanyl protein ligase, enhances sensitivity to CAMP (74), it is tempting to speculate that *L. crispatus* produces antimicrobial peptide(s) that kill *E. faecalis*.

In agreement with our speculation, we observed an upregulation of the *ompR-envZ* two-component system (*OG1RF_12162–63*), including *OG1RF_12161* directly downstream of *ompR-EnvZ*, that codes for an orphan putative D-Ala-D-Ala carboxypeptidase (Table 1; Table S1) in the RNAseq. In vancomycin-resistant enterococcal strains, the VanRS two-component system (TCS), which is a member of the OmpR-EnvZ two-component system (75), regulates a downstream vancomycin (Van) resistance accessory operon that is responsible for the modification of the terminal d-alanyl-d-alanine (d-Ala-d-Ala) dipeptide of the muramyl pentapeptide (76), a precursor in peptidoglycan synthesis, and in doing so, prevent the binding of Van and cell death. If indeed an antimicrobial peptide is facilitating *L. crispatus*-mediated killing, we anticipate resistance to killing in an *ompR/envZ*-responsive manner. Ironically, the *E. faecalis* OG1RF genome lacks a Van accessory operon. Paradoxically, *E. faecalis* V583, which is more susceptible to *L. crispatus*-mediated killing than OG1RF, possesses a Van accessory operon downstream of its VanRS_B_ (type B) TCS (*EF_2299–2298;* 32% and 22.4% a.a identity, respectively, to VanRS of OG1RF). Like V583 (77), another Van-resistant enterococcal strain, *E. faecalis* ATCC 51299 increased the synthesis of d-Ala-d-Lac-terminated peptidoglycan pentapeptides to prevent Van-mediated inhibition of peptidoglycan cross-linking (78). Perhaps, having more d-Ala-d-Lac-terminated pentapeptides renders *E. faecalis* V583 more vulnerable to *L crispatus*-mediated killing. Besides the fact that the coding sequences for a Zn-dependent D, D-carboxypeptidase (*OG1RF_12161*; 52% a.a) (annotated as *vanY* in V583 genome; for the cleavage of D-Ala-d-Ala dipeptides) is downstream of *ompR*/*envZ* in the core genome, studies of OmpR-facilitated remodeling of peptidoglycan in OG1RF have not been pursued. Ultimately, what we gathered from these findings is that the enterococcal cell wall interface seems to be the target of *L. crispatus* killing. While it is known that members of the lactobacilli family produce bacteriocins (79), among those are cell wall-targeting types (80), there have not been any studies indicating that *L. crispatus* does. However, based on genomic analysis, *L. crispatus* VPI 3199 genome encodes putative gene clusters for the synthesis of bacteriocins. Further studies are warranted to discern the underlying mechanisms.

Besides the *dltABCD* operon, *OG1RF_11697* was found to greatly contribute to *E. faecalis* fitness. Notably, *OG1RF_11697* codes for a hypothetical protein homologous to *L. monocytogenes* (37% similarity) and *S. aureus* (97% similarity) YbbR proteins. Like *L. monocytogenes* and *S. aureus*, *OG1RF_11697* (henceforth *ybbR*) resides in a conserved three-gene operon that includes the diadenylate cyclase CdaA (*OG1RF_11698*), and phosphoglucosamine mutase GlmM (*OG1RF_11696*). While the primary role of GlmM is to synthesize glucosamine-1-phosphate, an early metabolite intermediate for peptidoglycan synthesis; on the other hand, CdaA is the sole enzyme responsible for c-di-AMP synthesis in *E. faecalis* (81). While mechanisms remain poorly defined, recent studies have shown that YbbR and GlmM fine-tune intracellular c-di-AMP levels by enhancing or inhibiting the ability of CdaA to synthesize c-di-AMP. Specifically, co-expression of *S. aureus* CdaA with YbbR increased intracellular c-di-AMP pools, whereas co-expression of CdaA with GlmM reduced c-di-AMP levels (82). In *Lactococcus lactis*, a single amino acid substitution in GlmM resulted in a tighter interaction with CdaA and, as a result, triggered a drastic decrease in intracellular c-di-AMP levels (83). c-di-AMP regulates a wide range of cellular processes, including but not limited to osmoregulation, central metabolism, and virulence (84, 85). Because of the strong conservation of the *cdaA-ybbR-glmM* operon in Gram-positive bacteria, and multiple evidence associating YbbR and GlmM with c-di-AMP production, we speculate that the increased sensitivity of the *ybbR*::TnMar mutant to *L. crispatus* killing is linked to c-di-AMP production.

Unlike the decreased fitness observed in *dltABCD* and *ybbR* mutants, an explanation for the increased survival of *ldh1* mutants is less straightforward. One possibility is that the profound alteration in the fermentation end-product profile of Δ*ldh1* strains, due to the funneling of pyruvate to other enzymes in glycolysis, changing from mainly homolactic to heterolactic, that is, a distinct characteristic of the LDH-null strain that interferes with *L. crispatus* ability to produce anti-*E*. *faecalis* metabolites. Specifically, an *E. faecalis ∆ldh1∆ldh2* mutant that produces negligible amounts of lactate displays significant increases in formate (~4 fold), ethanol (~3 fold), and acetoin (~5 fold) produced (86). While the mechanism is unknown, both formate and ethanol have been shown to modulate the growth of lactobacilli. Specifically, co-culturing lactobacilli with *Saccharomyces cerevisiae* enhanced lactic acid production by lactobacilli while decreasing ethanol production by *S. cerevisiae* (87). In another example, formate produced by *Streptococcus thermophilus* stimulated *L. delbrueckii* growth and exopolysaccharide (EPS) production (88). Another possible, non-mutually exclusive explanation is that the metabolic alterations associated with the loss of LDH activity enhance *E. faecalis* overall fitness.

In summary, the findings presented here represent an important first step toward the identification of genetic determinants that allow *E. faecalis* to mitigate *L. crispatus* killing*,* which may have far-reaching implications to other ecological niches, that is, the gastrointestinal tract, where members of both genera coexist. In the long run, the findings here will provide explanations for the outgrowth of *E. faecalis* in the vaginal tract, and consequently, vaginal infection.

## MATERIALS AND METHODS

### Bacterial strains and growth conditions

The bacterial strains used in this study are listed in Table 3. Bacteria were grown in either brain heart infusion (BHI) broth (for enterococci) or de Man, Rogosa and Sharpe (MRS) broth (lactobacilli) (both from BD Difco, New Jersey, USA) at 37°C under microaerophilic conditions (5% CO_2_). For growth kinetic assays, overnight cultures were adjusted/normalized to an optical density (OD_600_) of 0.25 (for enterococci) or 0.75 (for lactobacilli) (~1 × 10^8^ CFU mL^−1^) and inoculated into fresh media at a 1:50 ratio, with changes in OD_600_ over time recorded in an automated growth reader (Bioscreen c, Oy Growth Curves AB, Helsinki, Finland). Unless stated otherwise, starter cultures for *in vitro* and *in vivo* experiments were adjusted from overnight cultures to the desired OD_600_ using phosphate-buffered saline (PBS). All biofilms were grown at 37°C under microaerophilic conditions (5% CO_2_) in MRS broth. For all mixed-species biofilms, enterococci and lactobacilli are mixed at a ratio of 1:1 prior to incubation. For *E. faecalis* OG1RF CFU determination, serially diluted aliquots were plated on BHI agar supplemented with 200 µg mL^−1^ rifampicin and 10 µg mL^−1^ fusidic acid. For CFU determination of other enterococci, bile aesculin azide (BAA) agar (BD Difco) was used. For lactobacilli CFU determination, serially diluted aliquots were plated on Rogosa agar (Oxoid, Massachusetts, USA). Chemicals and biological reagents were purchased from Sigma Aldrich (Missouri, USA) unless stated otherwise.

**TABLE 3:** Strains used in this study

	Strain name	Relevant characteristics^*a*^^,^^*b*^	References
*E. faecalis* strains	OG1RF parent strain (ATCC 47077)	Laboratory strain, Rif^R^, Fus^R^	(89)
	OG1RF ∆*ldh1*	Chromosomal deletion of *ldh1,* Rif^R^, Fus^R^	(27)
	OG1RF ∆*ldh1*∆*ldh2*	Chromosomal deletion of *ldh1* and *ldh2,* Rif^R^, Fus^R^	(99)
	OG1RF ∆*ldh1*::*ldh1*	Chromosomal integration of *ldh1,* Rif^R^, Fus^R^	(27)
	V583	Van^R^	(90)
	ST9 (WH257 MG1422)	Van^S^	(91)
*E. faecium* strains	59–06	Clinical isolate (laboratory collection)	This study
	ATCC 19634	ATCC Catalog	This study
*E. faecalis* OG1RF transposon mutants	*ldh1*::TnMar	Cm^R^, Erm^S^	(54)
	*dltA*::TnMar	Cm^R^, Erm^S^	(54)
	OG1RF_11697::TnMar	Cm^R^, Erm^S^	(54)
	OG1RF_12434::TnMar	Cm^R^, Erm^S^	(54)
	OG1RF_10490::TnMar	Cm^R^, Erm^S^	(54)
*L. crispatus* strains	VPI 3199 (Type strain)	ATCC collection	This study
	JV-V01 (BEI Cat. HM103)	Isolated from human vaginal flora^*a*^	This study^*a*^
	MV-1A-US (BEI Cat. HM637)	Isolated from human vaginal flora^*a*^	This study^*a*^
	MV-3A-US (BEI Cat. HM636)	Isolated from human vaginal flora^*a*^	This study^*a*^
	125–2-CHN (BEI Cat. HM638)	Isolated from human vaginal flora^*a*^	This study^*a*^
Other lactobacilli strains	*L. rhamnosus* GG	ATCC collection	This study
	*L. casei* ATCC 393 (type strain)	ATCC collection	This study

^*a*^ The following strains were obtained through BEI Resources, NIAID, and NIH as part of the Human Microbiome Project.

^*b*^ Resistant to kanamycin (Kan^R^), vancomycin (Van^R^), erythromycin (Erm^R^), gentamycin (Gen^R^), ampicillin (Amp^R^), streptomycin (Strep^R^), rifampicin (Rif^R^), fusidic acid (Fus^R^), or sensitive to chloramphenicol (Cm^s^) or vancomycin (Van^s^).

### Biofilm assay

Biofilm assays were performed as previously described with minor modifications (22). For macro-colony biofilm, 1 × 10^6^ CFU of each bacterium in a total volume of 5 µL was inoculated onto the surface of MRS agar. After incubation at selected timepoints, macro-colonies were excised using a scalpel and homogenized by vortexing in PBS, followed by plating on selective media for CFU enumeration. For single- and two-species biofilms established in broth, 1 × 10^6^ CFU of bacterial culture suspended in a total volume of 20 µL PBS was inoculated into 1 mL of MRS broth in a 24-well microtiter tissue culture plate. To investigate whether *L. crispatus*-mediated killing was contact-dependent or independent, biofilms were allowed to form in a six-well tissue culture-treated polystyrene plate with a Transwell insert (0.4 µm polyester membrane; Lot #20623011) (Corning, New York, USA). To investigate whether *L. crispatus*-secreted molecules are responsible for *E. faecalis* killing, biofilm supernatants (either single or dual-species biofilms grown for 72 h) were recovered and filter sterilized using 0.2 micron polyether sulfone membrane filters (Millipore, Massachusetts, USA), then mixed at equal ratio (vol/vol) with MRS media to grow *E. faecalis* biofilms.

### PLA susceptibility assay

Bacteria were grown overnight in MRS media, adjusted to an OD_600_ of 0.5 in PBS, and diluted at a ratio of 1:1,000. Diluted cultures were inoculated at a ratio of 1:20 into MRS broth containing increasing concentrations of phenyl-lactic acid and incubated at 37°C with 5% CO_2_ for 24  h. The absorbance at OD_600_ was measured using a Synergy H1 microplate reader (Molecular Devices, California, USA).

### Spot antagonism assay

Bacterial spot biofilms were prepared as previously described (92) with minor modifications. Briefly, 10 µL of normalized starter cultures was spotted on MRS plates with *E. faecalis* inoculated at the same time or 24, 48, or 72 h after *L. crispatus*.

### *Galleria mellonella* infection

To assess *E. faecalis* virulence, the larvae of *G. mellonella* were infected as described previously (89). Briefly, larvae (groups of 20) were injected with (i) *E. faecalis* OG1RF or *L. crispatus* VPI 3199 alone (~ 5 x 10^5^ CFU), (ii) heat-killed OG1RF or VPI3199 (60 min at 100°C), or (iii) dual-species inoculum. Post-injection, larvae were kept at 37°C, and their survival was recorded over time for 72 h.

### Transposon mutant library growth as dual species macro-colony biofilms

The *E. faecalis* OG1RF pooled Tn library (52) was recovered from glycerol stock and suspended in 1 mL of PBS. Single-species *E. faecalis* pooled Tn library and *L. crispatus* macro-colony biofilms were produced by inoculating 1 × 10^6^ CFU of each in a total volume of 5 µL onto the surface of MRS agar. To produce dual-species macro-colony biofilms, an equal volume of single-species *E. faecalis* pooled transposon library and *L. crispatus* were mixed and inoculated onto the surface of MRS agar. After incubation at selected timepoints, macrocolonies were excised using a scalpel and suspended in PBS. For Tn-seq, single-species and dual-species macrocolony biofilm samples were prepared accordingly, then pelleted at 4,000 × rpm for 10 min, and frozen at −80°C until further use.

### Tn-seq analysis

The Wizard DNA purification kit (Promega, Wisconsin, USA) was used to obtain genomic DNA from single-species or dual-species macro-colony pellets (prepared in triplicates). Samples were submitted to the University of Minnesota Genomics Center for library preparation and sequencing. Sequencing was performed using an Illumina NextSeq 2000 in 150 bp paired-end output mode. Tn-seq analysis was performed with custom scripts using the University of Florida supercomputer HiperGator. Analysis of sequencing reads was performed as previously described (93). Briefly, bioinformatics reads were mapped to the OG1RF reference genome (GenBank CP002621.1, NCBI RefSeq NC_017316.1), and Tn insertions at TA sites were quantified. The statistical significance of the relative abundance of Tn reads at each TA site in each corresponding single-species biofilm compared to dual-species biofilm samples was evaluated using a chi-square test and a Monte Carlo-based method. Th scripts used in this study are publicly available (https://github.com/dunnylabumn/Ef_OG1RF_TnSeq). Log_2_ fold changes were calculated from each corresponding single-species biofilm compared to dual-species biofilm samples. Statistical significance was defined as a *P* value of <0.05 and a Monte Carlo simulation value of 3.73184 (the lowest value obtained in these calculations). Tn mutants used for additional experiments were obtained from frozen library stock plates (gifts from Dunny lab, University of Minnesota, USA). Verification of Tn mutants was performed as previously described using selective plating and sequencing (54, 94).

### RNA sequencing and analysis

Triplicates of single-species *E. faecalis* static biofilms were produced by inoculating 1 × 10^6^ CFU in a total volume of 5 mL MRS media in a six-well tissue culture plate. To produce co-culture biofilms, equal inocula of single-species *E. faecalis* and *L. crispatus* cells were inoculated into the same well of a tissue culture plate. Separated by a Transwell (Corning, New York, USA) permeable membrane insert that prevents physical, *E. faecalis* biofilms were seeded on the surface of the Transwell permeable membrane, whereas *L. crispatus* biofilms were grown on the bottom of the tissue culture plate. After incubation for 24 h, biofilm samples were pelleted at 4,000 × rpm for 10 min, then suspended in RNAprotect solution (Qiagen, Hilden, Germany) for 5 min. Then, samples were pelleted at 4,000 × rpm for 10 min again for downstream RNA extraction. Subsequent RNA extraction steps were performed according to the manufacturer’s protocol using PureLink RNA mini kit (Thermofisher, Massachusetts, USA) with minor modifications. Briefly, samples were incubated with 30 mg mL^−1^ of lysozyme for 30 min prior to extracting RNA. After extraction, RNA samples were further processed using the Monarch RNA cleanup kit (New England Biolabs, Massachusetts, USA). Then, the extracted RNA was quality-checked using the RNA ScreenTape on a TapeStation instrument (Agilent Technologies, USA). Every sample had a minimum RNA concentration of 25 ng/µL and a RINe value ≥8.0. Extracted RNA samples were sent to SeqCenter (Pennsylvania, USA) for library preparation, ribosomal RNA depletion, sequencing, and downstream bioinformatic analysis. Library preparation was performed using Illumina’s Stranded Total RNA Prep Ligation with Ribo-Zero Plus kit and 10 bp unique dual indices (UDI). Sequencing was done on a NovaSeq X Plus, producing paired-end 150 bp reads. Demultiplexing, quality control, and adapter trimming were performed with Illumina software bcl-convert (v4.2.4). Read mapping was performed with HISAT2 (95). Read quantification was performed using Subread’s featureCounts (96). Read counts were normalized using edgeR’s Trimmed Mean of M values (TMM) algorithm (97). Subsequent values were then converted to counts per million (CPM). Differential expression analysis was performed using edgeR’s glmQLFTest. Differentially expressed genes were considered using a cut-off of log_2_ fold change (between ≤−1 and ≥1) and *P* ≤ 0.05. Finally, pathway analysis was performed using limma’s *“kegga”* functionality (98). The genes that were considered Up/Down in this analysis were set at a cut-off using Benjamini-Hochberg adjusted *P* ≤ 0.05 (−*logFDR* of 1.3).

### Statistical analysis

Data obtained from this study were analyzed using GraphPad Prism 9.0 software (GraphPad Software, San Diego, California, USA). Data from multiple experiments conducted on non-consecutive days were collated and applicable statistical tests were used.

## Data Availability

The authors confirm that the data supporting the findings of this study are available within the article or in the supplementary material. The sequencing files generated from Tn-Seq (BioProject ID: PRJNA1170924) and RNA-seq (GEO Accession ID: GSE279275) have been deposited with the NCBI Sequence Read Archive and Gene Expression Omnibus database.

## AFTER EPUB

[This article was published on 4 February 2025 with errors in Acknowledgments and Table 3. The errors were corrected in the current version, posted on 10 March 2025.]

## SUPPLEMENTAL MATERIAL

Supplemental figures jb.00438-24-s0001.docx Fig. S1 to S6.

Table S1 jb.00438-24-s0002.xlsx RNAseq data.

Table S2 jb.00438-24-s0003.xlsx KEGG analysis of RNAseq analysis.

Table S3 jb.00438-24-s0004.xlsx Tnseq comparison data at 24hr.

Table S4 jb.00438-24-s0005.xlsx Tnseq comparison data at 48hr.

Table S5 jb.00438-24-s0006.xlsx Tnseq comparison data at 72hr.

ASM does not own the copyrights to Supplemental Material that may be linked to, or accessed through, an article. The authors have granted ASM a non-exclusive, world-wide license to publish the Supplemental Material files. Please contact the corresponding author directly for reuse.

## References

[1] Flores-Mireles AL, Walker JN, Caparon M, Hultgren SJ. 2015. Urinary tract infections: epidemiology, mechanisms of infection and treatment options. Nat Rev Microbiol 13:269–284. doi:10.1038/nrmicro3432 25853778 PMC4457377

[2] Ubeda C, Taur Y, Jenq RR, Equinda MJ, Son T, Samstein M, Viale A, Socci ND, van den Brink MRM, Kamboj M, Pamer EG. 2010. Vancomycin-resistant Enterococcus domination of intestinal microbiota is enabled by antibiotic treatment in mice and precedes bloodstream invasion in humans. J Clin Invest 120:4332–4341. doi:10.1172/JCI43918 21099116 PMC2993598

[3] Chanderraj R, Brown CA, Hinkle K, Falkowski N, Ranjan P, Dickson RP, Woods RJ. 2020. Gut microbiota predict Enterococcus expansion but not vancomycin-resistant Enterococcus acquisition. mSphere 5:e00537-20. doi:10.1128/msphere.00537-20 33208515 PMC7677005

[4] Perzon O, Cahn A, Gellman YN, Leibovitch M, Peled S, Elishoov O, Haze A, Olshtain-Pops K, Elinav H. 2023. Enterococci in diabetic foot infections: prevalence, clinical characteristics, and outcomes. Open Forum Infect Dis 10:ofad238. doi:10.1093/ofid/ofad238 37234514 PMC10205551

[5] Alghamdi F, Shakir M. 2020. The influence of Enterococcus faecalis as a dental root canal pathogen on endodontic treatment: a systematic review. Cureus 12:e7257. doi:10.7759/cureus.7257 32292671 PMC7152576

[6] Herrera-Hidalgo L, Fernández-Rubio B, Luque-Márquez R, López-Cortés LE, Gil-Navarro MV, de Alarcón A. 2023. Treatment of Enterococcus faecalis infective endocarditis: a continuing challenge. Antibiotics (Basel) 12:704. doi:10.3390/antibiotics12040704 37107066 PMC10135260

[7] Nye C, Maxwell A, Hughes H, Underwood J. 2024. Enterococcus faecalis bacteraemia and infective endocarditis - what are we missing? Clin Infect Pract 21:100336. doi:10.1016/j.clinpr.2023.100336 38404506 PMC7615663

[8] Werneburg GT. 2022. Catheter-associated urinary tract infections: current challenges and future prospects. Res Rep Urol 14:109–133. doi:10.2147/RRU.S273663 35402319 PMC8992741

[9] Farsi S, Salama I, Escalante-Alderete E, Cervantes J. 2023. Multidrug-resistant enterococcal infection in surgical patients, what surgeons need to know. Microorganisms 11:238. doi:10.3390/microorganisms11020238 36838203 PMC9968095

[10] Amat-Santos IJ, Ribeiro HB, Urena M, Allende R, Houde C, Bédard E, Perron J, DeLarochellière R, Paradis JM, Dumont E, Doyle D, Mohammadi S, Côté M, San Roman JA, Rodés-Cabau J. 2015. Prosthetic valve endocarditis after transcatheter valve replacement: a systematic review. JACC Cardiovasc Interv 8:334–346. doi:10.1016/j.jcin.2014.09.013 25700757

[11] Arias CA, Murray BE. 2012. The rise of the Enterococcus: beyond vancomycin resistance. Nat Rev Microbiol 10:266–278. doi:10.1038/nrmicro2761 22421879 PMC3621121

[12] Gaca AO, Lemos JA. 2019. Adaptation to adversity: the intermingling of stress tolerance and pathogenesis in enterococci. Microbiol Mol Biol Rev 83:e00008-19. doi:10.1128/MMBR.00008-19 31315902 PMC6710459

[13] Ch’ng J-H, Chong KKL, Lam LN, Wong JJ, Kline KA. 2019. Biofilm-associated infection by enterococci. Nat Rev Microbiol 17:82–94. doi:10.1038/s41579-018-0107-z 30337708 PMC13729018

[14] Șchiopu P, Toc DA, Colosi IA, Costache C, Ruospo G, Berar G, Gălbău Ștefan-G, Ghilea AC, Botan A, Pană A-G, Neculicioiu VS, Todea DA. 2023. An overview of the factors involved in biofilm production by the Enterococcus genus. Int J Mol Sci 24:11577. doi:10.3390/ijms241411577 37511337 PMC10380289

[15] Michaelis C, Grohmann E. 2023. Horizontal gene transfer of antibiotic resistance genes in biofilms. Antibiotics (Basel) 12:328. doi:10.3390/antibiotics12020328 36830238 PMC9952180

[16] Sharma S, Mohler J, Mahajan SD, Schwartz SA, Bruggemann L, Aalinkeel R. 2023. Microbial biofilm: a review on formation, infection, antibiotic resistance, control measures, and innovative treatment. Microorganisms 11:1614. doi:10.3390/microorganisms11061614 37375116 PMC10305407

[17] Kulshrestha A, Gupta P. 2022. Polymicrobial interaction in biofilm: mechanistic insights. Pathog Dis 80:ftac010. doi:10.1093/femspd/ftac010 35481543

[18] Han SK, Shin MS, Park HE, Kim SY, Lee WK. 2014. Screening of bacteriocin-producing Enterococcus faecalis strains for antagonistic activities against Clostridium perfringens. Korean J Food Sci Anim Resour 34:614–621. doi:10.5851/kosfa.2014.34.5.614 26761495 PMC4662223

[19] Krüger M, Shehata AA, Schrödl W, Rodloff A. 2013. Glyphosate suppresses the antagonistic effect of Enterococcus spp. on Clostridium botulinum. Anaerobe 20:74–78. doi:10.1016/j.anaerobe.2013.01.005 23396248

[20] Suzuki N, Yoneda M, Hatano Y, Iwamoto T, Masuo Y, Hirofuji T. 2011. Enterococcus faecium WB2000 inhibits biofilm formation by oral cariogenic streptococci. Int J Dent 2011:834151. doi:10.1155/2011/834151 22114599 PMC3205667

[21] Graham CE, Cruz MR, Garsin DA, Lorenz MC. 2017. Enterococcus faecalis bacteriocin EntV inhibits hyphal morphogenesis, biofilm formation, and virulence of Candida albicans Proc Natl Acad Sci U S A 114:4507–4512. doi:10.1073/pnas.1620432114 28396417 PMC5410809

[22] Keogh D, Tay WH, Ho YY, Dale JL, Chen S, Umashankar S, Williams RBH, Chen SL, Dunny GM, Kline KA. 2016. Enterococcal metabolite cues facilitate interspecies niche modulation and polymicrobial infection. Cell Host Microbe 20:493–503. doi:10.1016/j.chom.2016.09.004 27736645 PMC5076562

[23] Laganenka L, Sourjik V. 2018. Autoinducer 2-dependent Escherichia coli biofilm formation is enhanced in a dual-species coculture. Appl Environ Microbiol 84:e02638-17. doi:10.1128/AEM.02638-17 29269492 PMC5812939

[24] Tien BYQ, Goh HMS, Chong KKL, Bhaduri-Tagore S, Holec S, Dress R, Ginhoux F, Ingersoll MA, Williams RBH, Kline KA. 2017. Enterococcus faecalis promotes innate immune suppression and polymicrobial catheter-associated urinary tract infection. Infect Immun 85:e00378-17. doi:10.1128/IAI.00378-17 28893918 PMC5695114

[25] Phan S, Feng CH, Huang R, Lee ZX, Moua Y, Phung OJ, Lenhard JR. 2023. Relative abundance and detection of Pseudomonas aeruginosa from chronic wound infections globally. Microorganisms 11:1210. doi:10.3390/microorganisms11051210 37317184 PMC10222620

[26] Lee K, Lee KM, Kim D, Yoon SS. 2017. Molecular determinants of the thickened matrix in a dual-species Pseudomonas aeruginosa and Enterococcus faecalis biofilm. Appl Environ Microbiol 83:e01182-17. doi:10.1128/AEM.01182-17 28842537 PMC5648906

[27] Tan CAZ, Lam LN, Biukovic G, Soh EY-C, Toh XW, Lemos JA, Kline KA. 2022. Enterococcus faecalis antagonizes Pseudomonas aeruginosa growth in mixed-species interactions. J Bacteriol 204:e0061521. doi:10.1128/jb.00615-21 35758750 PMC9295543

[28] Smith AB, Jenior ML, Keenan O, Hart JL, Specker J, Abbas A, Rangel PC, Di C, Green J, Bustin KA, et al.. 2022. Enterococci enhance Clostridioides difficile pathogenesis. Nature New Biol 611:780–786. doi:10.1038/s41586-022-05438-x PMC9691601 36385534

[29] Ch’ng J-H, Muthu M, Chong KKL, Wong JJ, Tan CAZ, Koh ZJS, Lopez D, Matysik A, Nair ZJ, Barkham T, Wang Y, Kline KA. 2022. Heme cross-feeding can augment Staphylococcus aureus and Enterococcus faecalis dual species biofilms. ISME J 16:2015–2026. doi:10.1038/s41396-022-01248-1 35589966 PMC9296619

[30] Kang MS, Lim HS, Oh JS, Lim YJ, Wuertz-Kozak K, Harro JM, Shirtliff ME, Achermann Y. 2017. Antimicrobial activity of Lactobacillus salivarius and Lactobacillus fermentum against Staphylococcus aureus. Pathog Dis 75. doi:10.1093/femspd/ftx009 28158586

[31] Jamalifar H, Rahimi H, Samadi N, Shahverdi A, Sharifian Z, Hosseini F, Eslahi H, Fazeli M. 2011. Antimicrobial activity of different Lactobacillus species against multi- drug resistant clinical isolates of Pseudomonas aeruginosa. Iran J Microbiol 3:21–25. 22347578 PMC3279796

[32] Chen C-C, Lai C-C, Huang H-L, Huang W-Y, Toh H-S, Weng T-C, Chuang Y-C, Lu Y-C, Tang H-J. 2019. Antimicrobial activity of Lactobacillus species against carbapenem-resistant Enterobacteriaceae. Front Microbiol 10. doi:10.3389/fmicb.2019.00789 PMC6482263 31057508

[33] Di Cerbo A, Palmieri B, Aponte M, Morales-Medina JC, Iannitti T. 2016. Mechanisms and therapeutic effectiveness of lactobacilli. J Clin Pathol 69:187–203. doi:10.1136/jclinpath-2015-202976 26578541 PMC4789713

[34] Ravel J, Gajer P, Abdo Z, Schneider GM, Koenig SSK, McCulle SL, Karlebach S, Gorle R, Russell J, Tacket CO, Brotman RM, Davis CC, Ault K, Peralta L, Forney LJ. 2011. Vaginal microbiome of reproductive-age women. Proc Natl Acad Sci U S A 108 Suppl 1:4680–4687. doi:10.1073/pnas.1002611107 20534435 PMC3063603

[35] France MT, Ma B, Gajer P, Brown S, Humphrys MS, Holm JB, Waetjen LE, Brotman RM, Ravel J. 2020. VALENCIA: a nearest centroid classification method for vaginal microbial communities based on composition. Microbiome 8:166. doi:10.1186/s40168-020-00934-6 33228810 PMC7684964

[36] Auriemma RS, Scairati R, Del Vecchio G, Liccardi A, Verde N, Pirchio R, Pivonello R, Ercolini D, Colao A. 2021. The vaginal microbiome: a long urogenital colonization throughout woman life. Front Cell Infect Microbiol 11:686167. doi:10.3389/fcimb.2021.686167 34295836 PMC8290858

[37] Chen X, Lu Y, Chen T, Li R. 2021. The female vaginal microbiome in health and bacterial vaginosis. Front Cell Infect Microbiol 11. doi:10.3389/fcimb.2021.631972 PMC8058480 33898328

[38] Chee WJY, Chew SY, Than LTL. 2020. Vaginal microbiota and the potential of Lactobacillus derivatives in maintaining vaginal health. Microb Cell Fact 19:203. doi:10.1186/s12934-020-01464-4 33160356 PMC7648308

[39] Jahic M. 2022. Aerobic vaginitis caused by Enterococcus faecalis - clinical features and treatment. Mater Sociomed 34:291–295. doi:10.5455/msm.2022.34.291-295 36936892 PMC10019880

[40] Donders GGG, Bellen G, Grinceviciene S, Ruban K, Vieira-Baptista P. 2017. Aerobic vaginitis: no longer a stranger. Res Microbiol 168:845–858. doi:10.1016/j.resmic.2017.04.004 28502874

[41] Khan I, Khan UA. 2004. A hospital based study of frequency of aerobic pathogens in vaginal infections. RMJ 29:22–25.

[42] Sangeetha KT, Golia S, Vasudha CL. 2017. A study of aerobic bacterial pathogens associated with vaginitis in reproductive age group women (15-45 years) and their sensitivity pattern. Int J Res Med Sci 3:2268–2273. doi:10.18203/2320-6012.ijrms20150615

[43] Alhajjar N, Chatterjee A, Spencer BL, Burcham LR, Willett JLE, Dunny GM, Duerkop BA, Doran KS. 2020. Genome-wide mutagenesis identifies factors involved in Enterococcus faecalis vaginal adherence and persistence. Infect Immun 88:e00270-20. doi:10.1128/IAI.00270-20 32778611 PMC7504943

[44] Mondal AS, Sharma R, Trivedi N. 2023. Bacterial vaginosis: a state of microbial dysbiosis. Med Microecol 16:100082. doi:10.1016/j.medmic.2023.100082

[45] Amabebe E, Anumba DOC. 2018. The vaginal microenvironment: the physiologic role of lactobacilli Front Med (Lausanne) 5:181. doi:10.3389/fmed.2018.00181 29951482 PMC6008313

[46] Lepargneur JP. 2016. Lactobacillus crispatus as biomarker of the healthy vaginal tract. Ann Biol Clin (Paris) 74:421–427. doi:10.1684/abc.2016.1169 27492695

[47] Abdul-Rahim O, Wu Q, Price TK, Pistone G, Diebel K, Bugni TS, Wolfe AJ. 2021. Phenyl-lactic acid is an active ingredient in bactericidal supernatants of Lactobacillus crispatus. J Bacteriol 203:e0036021. doi:10.1128/JB.00360-21 34280003 PMC8425402

[48] Wang F, Wu H, Jin P, Sun Z, Liu F, Du L, Wang D, Xu W. 2018. Antimicrobial activity of phenyllactic acid against Enterococcus faecalis and its effect on cell membrane. Foodborne Pathog Dis 15:645–652. doi:10.1089/fpd.2018.2470 30227085

[49] Valerio F, Lavermicocca P, Pascale M, Visconti A. 2004. Production of phenyllactic acid by lactic acid bacteria: an approach to the selection of strains contributing to food quality and preservation. FEMS Microbiol Lett 233:289–295. doi:10.1016/j.femsle.2004.02.020 15063498

[50] Jung S, Hwang H, Lee J-H. 2019. Effect of lactic acid bacteria on phenyllactic acid production in kimchi. Food Control 106:106701. doi:10.1016/j.foodcont.2019.06.027

[51] Tachedjian G, Aldunate M, Bradshaw CS, Cone RA. 2017. The role of lactic acid production by probiotic Lactobacillus species in vaginal health. Res Microbiol 168:782–792. doi:10.1016/j.resmic.2017.04.001 28435139

[52] Dale JL, Beckman KB, Willett JLE, Nilson JL, Palani NP, Baller JA, Hauge A, Gohl DM, Erickson R, Manias DA, Sadowsky MJ, Dunny GM. 2018. Comprehensive functional analysis of the Enterococcus faecalis core genome using an ordered, sequence-defined collection of insertional mutations in strain OG1RF. mSystems 3:e00062-18. doi:10.1128/mSystems.00062-18 30225373 PMC6134198

[53] Nguyen AH, Hood KS, Mileykovskaya E, Miller WR, Tran TT. 2022. Bacterial cell membranes and their role in daptomycin resistance: a review. Front Mol Biosci 9. doi:10.3389/fmolb.2022.1035574 PMC9702088 36452455

[54] Kristich CJ, Nguyen VT, Le T, Barnes AMT, Grindle S, Dunny GM. 2008. Development and use of an efficient system for random mariner transposon mutagenesis to identify novel genetic determinants of biofilm formation in the core Enterococcus faecalis genome. Appl Environ Microbiol 74:3377–3386. doi:10.1128/AEM.02665-07 18408066 PMC2423031

[55] Keogh D, Lam LN, Doyle LE, Matysik A, Pavagadhi S, Umashankar S, Low PM, Dale JL, Song Y, Ng SP, Boothroyd CB, Dunny GM, Swarup S, Williams RBH, Marsili E, Kline KA. 2018. Extracellular electron transfer powers Enterococcus faecalis biofilm metabolism. MBio 9:e00626-17. doi:10.1128/mBio.00626-17 PMC5893876 29636430

[56] Rana NF, Sauvageot N, Laplace JM, Bao Y, Nes I, Rincé A, Posteraro B, Sanguinetti M, Hartke A. 2013. Redox balance via lactate dehydrogenase is important for multiple stress resistance and virulence in Enterococcus faecalis. Infect Immun 81:2662–2668. doi:10.1128/IAI.01299-12 23649090 PMC3719593

[57] Leboeuf C, Leblanc L, Auffray Y, Hartke A. 2000. Characterization of the ccpA gene of Enterococcus faecalis: identification of starvation-inducible proteins regulated by ccpA. J Bacteriol 182:5799–5806. doi:10.1128/JB.182.20.5799-5806.2000 11004180 PMC94703

[58] Walter J. 2008. Ecological role of lactobacilli in the gastrointestinal tract: implications for fundamental and biomedical research. Appl Environ Microbiol 74:4985–4996. doi:10.1128/AEM.00753-08 18539818 PMC2519286

[59] Colautti A, Orecchia E, Comi G, Iacumin L. 2022. Lactobacilli, a weapon to counteract pathogens through the inhibition of their virulence factors. J Bacteriol 204:e0027222. doi:10.1128/jb.00272-22 36286515 PMC9664955

[60] Dempsey E, Corr SC. 2022. Lactobacillus spp. for gastrointestinal health: current and future perspectives. Front Immunol 13:840245. doi:10.3389/fimmu.2022.840245 35464397 PMC9019120

[61] Liu P, Lu Y, Li R, Chen X. 2023. Use of probiotic lactobacilli in the treatment of vaginal infections: in vitro and in vivo investigations. Front Cell Infect Microbiol 13:1153894. doi:10.3389/fcimb.2023.1153894 37077531 PMC10106725

[62] Huang R, Wu F, Zhou Q, Wei W, Yue J, Xiao B, Luo Z. 2022. Lactobacillus and intestinal diseases: mechanisms of action and clinical applications. Microbiol Res 260:127019. doi:10.1016/j.micres.2022.127019 35421680

[63] Paganelli FL, Willems RJ, Leavis HL. 2012. Optimizing future treatment of enterococcal infections: attacking the biofilm? Trends Microbiol 20:40–49. doi:10.1016/j.tim.2011.11.001 22169461

[64] Ma X, Wu M, Wang C, Li H, Fan A, Wang Y, Han C, Xue F. 2022. The pathogenesis of prevalent aerobic bacteria in aerobic vaginitis and adverse pregnancy outcomes: a narrative review. Reprod Health 19:21. doi:10.1186/s12978-021-01292-8 35090514 PMC8796570

[65] Amabebe E, Bhatnagar N, Kamble N, Reynolds S, Anumba DO. 2022. Exploring the antimicrobial properties of vaginal Lactobacillus crispatus against preterm birth-associated bacteria. Reprod Fertil 3:L6–L8. doi:10.1530/RAF-22-0026 35928673 PMC9346311

[66] Jang SJ, Lee K, Kwon B, You HJ, Ko G. 2019. Vaginal lactobacilli inhibit growth and hyphae formation of Candida albicans. Sci Rep 9:8121. doi:10.1038/s41598-019-44579-4 31148560 PMC6544633

[67] Mejia ME, Robertson CM, Patras KA. 2023. Interspecies interactions within the host: the social network of group B Streptococcus Infect Immun 91:e0044022. doi:10.1128/iai.00440-22 36975791 PMC10112235

[68] Marziali G, Foschi C, Parolin C, Vitali B, Marangoni A. 2019. In-vitro effect of vaginal lactobacilli against group B Streptococcus. Microb Pathog 136:103692. doi:10.1016/j.micpath.2019.103692 31445119

[69] Shiroda M, Aronoff DM, Gaddy JA, Manning SD. 2020. The impact of Lactobacillus on group B streptococcal interactions with cells of the extraplacental membranes. Microb Pathog 148:104463. doi:10.1016/j.micpath.2020.104463 32828901 PMC7683368

[70] He Y, Niu X, Wang B, Na R, Xiao B, Yang H. 2020. Evaluation of the inhibitory effects of Lactobacillus gasseri and Lactobacillus crispatus on the adhesion of seven common lower genital tract infection-causing pathogens to vaginal epithelial cells. Front Med 7:284. doi:10.3389/fmed.2020.00284 PMC7317292 32637420

[71] Egan AJF, Errington J, Vollmer W. 2020. Regulation of peptidoglycan synthesis and remodelling. Nat Rev Microbiol 18:446–460. doi:10.1038/s41579-020-0366-3 32424210

[72] Percy MG, Gründling A. 2014. Lipoteichoic acid synthesis and function in gram-positive bacteria. Annu Rev Microbiol 68:81–100. doi:10.1146/annurev-micro-091213-112949 24819367

[73] Neuhaus FC, Baddiley J. 2003. A continuum of anionic charge: structures and functions of D-alanyl-teichoic acids in gram-positive bacteria. Microbiol Mol Biol Rev 67:686–723. doi:10.1128/MMBR.67.4.686-723.2003 14665680 PMC309049

[74] Fabretti F, Theilacker C, Baldassarri L, Kaczynski Z, Kropec A, Holst O, Huebner J. 2006. Alanine esters of enterococcal lipoteichoic acid play a role in biofilm formation and resistance to antimicrobial peptides. Infect Immun 74:4164–4171. doi:10.1128/IAI.00111-06 16790791 PMC1489678

[75] Kenney LJ, Anand GS. 2020. EnvZ/OmpR two-component signaling: an archetype system that can function noncanonically. EcoSal Plus 9. doi:10.1128/ecosalplus.ESP-0001-2019 PMC7192543 32003321

[76] Guffey AA, Loll PJ. 2021. Regulation of resistance in vancomycin-resistant enterococci: the VanRS two-component system. Microorganisms 9:2026. doi:10.3390/microorganisms9102026 34683347 PMC8541618

[77] Evers S, Courvalin P. 1996. Regulation of VanB-type vancomycin resistance gene expression by the VanS_B_-VanR_B_ two-component regulatory system in Enterococcus faecalis V583. J Bacteriol 178:1302–1309. doi:10.1128/jb.178.5.1302-1309.1996 8631706 PMC177803

[78] Chang JD, Foster EE, Yang H, Kim SJ. 2017. Quantification of the d-Ala-d-Lac-terminated peptidoglycan structure in vancomycin-resistant Enterococcus faecalis using a combined solid-state nuclear magnetic resonance and mass spectrometry analysis. Biochemistry 56:612–622. doi:10.1021/acs.biochem.6b00774 28040891 PMC6906607

[79] Collins FWJ, O’Connor PM, O’Sullivan O, Gómez-Sala B, Rea MC, Hill C, Ross RP. 2017. Bacteriocin gene-trait matching across the complete Lactobacillus pan-genome. Sci Rep 7:3481. doi:10.1038/s41598-017-03339-y 28615683 PMC5471241

[80] Roces C, Rodríguez A, Martínez B. 2012. Cell wall-active bacteriocins and their applications beyond antibiotic activity. Probiotics Antimicrob Proteins 4:259–272. doi:10.1007/s12602-012-9116-9 26782186

[81] Kundra S, Lam LN, Kajfasz JK, Casella LG, Andersen MJ, Abranches J, Flores-Mireles AL, Lemos JA. 2021. c-di-AMP is essential for the virulence of Enterococcus faecalis Infect Immun 89:e0036521. doi:10.1128/IAI.00365-21 34424750 PMC8519298

[82] Tosi T, Hoshiga F, Millership C, Singh R, Eldrid C, Patin D, Mengin-Lecreulx D, Thalassinos K, Freemont P, Gründling A. 2019. Inhibition of the Staphylococcus aureus c-di-AMP cyclase DacA by direct interaction with the phosphoglucosamine mutase GlmM. PLoS Pathog 15:e1007537. doi:10.1371/journal.ppat.1007537 30668586 PMC6368335

[83] Zhu Y, Pham TH, Nhiep THN, Vu NMT, Marcellin E, Chakrabortti A, Wang Y, Waanders J, Lo R, Huston WM, Bansal N, Nielsen LK, Liang Z-X, Turner MS. 2016. Cyclic-di-AMP synthesis by the diadenylate cyclase CdaA is modulated by the peptidoglycan biosynthesis enzyme GlmM in Lactococcus lactis. Mol Microbiol 99:1015–1027. doi:10.1111/mmi.13281 26585449

[84] He J, Yin W, Galperin MY, Chou S-H. 2020. Cyclic di-AMP, a second messenger of primary importance: tertiary structures and binding mechanisms. Nucleic Acids Res 48:2807–2829. doi:10.1093/nar/gkaa112 32095817 PMC7102992

[85] Zarrella TM, Bai G. 2020. The many roles of the bacterial second messenger cyclic di-AMP in adapting to stress cues. J Bacteriol 203:e00348-20. doi:10.1128/JB.00348-20 32839175 PMC7723955

[86] Jönsson M, Saleihan Z, Nes IF, Holo H. 2009. Construction and characterization of three lactate dehydrogenase-negative Enterococcus faecalis V583 mutants. Appl Environ Microbiol 75:4901–4903. doi:10.1128/AEM.00344-09 19465534 PMC2708445

[87] Narendranath NV, Hynes SH, Thomas KC, Ingledew WM. 1997. Effects of lactobacilli on yeast-catalyzed ethanol fermentations. Appl Environ Microbiol 63:4158–4163. doi:10.1128/aem.63.11.4158-4163.1997 9361399 PMC168732

[88] Nishimura J, Kawai Y, Aritomo R, Ito Y, Makino S, Ikegami S, Isogai E, Saito T. 2013. Effect of formic acid on exopolysaccharide production in skim milk fermentation by Lactobacillus delbrueckii subsp. bulgaricus OLL1073R-1. Biosci Microbiota Food Health 32:23–32. doi:10.12938/bmfh.32.23 24936359 PMC4034293

[89] Lam LN, Brunson DN, Molina JJ, Flores-Mireles AL, Lemos JA. 2022. The AdcACB/AdcAII system is essential for zinc homeostasis and an important contributor of Enterococcus faecalis virulence. Virulence 13:592–608. doi:10.1080/21505594.2022.2056965 35341449 PMC8966984

[90] Sahm DF, Kissinger J, Gilmore MS, Murray PR, Mulder R, Solliday J, Clarke B. 1989. In vitro susceptibility studies of vancomycin-resistant Enterococcus faecalis. Antimicrob Agents Chemother 33:1588–1591. doi:10.1128/AAC.33.9.1588 2554802 PMC172707

[91] McBride SM, Fischetti VA, Leblanc DJ, Moellering RC Jr, Gilmore MS. 2007. Genetic diversity among Enterococcus faecalis. PLoS One 2:e582. doi:10.1371/journal.pone.0000582 17611618 PMC1899230

[92] Zeng L, Walker AR, Burne RA, Taylor ZA. 2023. Glucose phosphotransferase system modulates pyruvate metabolism, bacterial fitness, and microbial ecology in oral streptococci. J Bacteriol 205:e0035222. doi:10.1128/jb.00352-22 36468868 PMC9879115

[93] Willett JLE, Dale JL, Kwiatkowski LM, Powers JL, Korir ML, Kohli R, Barnes AMT, Dunny GM. 2021. Comparative biofilm assays using Enterococcus faecalis OG1RF identify new determinants of biofilm formation. MBio 12:e0101121. doi:10.1128/mBio.01011-21 34126766 PMC8262879

[94] Kristich CJ, Chandler JR, Dunny GM. 2007. Development of a host-genotype-independent counterselectable marker and a high-frequency conjugative delivery system and their use in genetic analysis of Enterococcus faecalis. Plasmid 57:131–144. doi:10.1016/j.plasmid.2006.08.003 16996131 PMC1852458

[95] Kim D, Paggi JM, Park C, Bennett C, Salzberg SL. 2019. Graph-based genome alignment and genotyping with HISAT2 and HISAT-genotype. Nat Biotechnol 37:907–915. doi:10.1038/s41587-019-0201-4 31375807 PMC7605509

[96] Liao Y, Smyth GK, Shi W. 2014. featureCounts: an efficient general purpose program for assigning sequence reads to genomic features. Bioinformatics 30:923–930. doi:10.1093/bioinformatics/btt656 24227677

[97] Robinson MD, McCarthy DJ, Smyth GK. 2010. edgeR: a bioconductor package for differential expression analysis of digital gene expression data. Bioinformatics 26:139–140. doi:10.1093/bioinformatics/btp616 19910308 PMC2796818

[98] Ritchie ME, Phipson B, Wu D, Hu Y, Law CW, Shi W, Smyth GK. 2015. limma powers differential expression analyses for RNA-sequencing and microarray studies. Nucleic Acids Res 43:e47. doi:10.1093/nar/gkv007 25605792 PMC4402510

[99] da Silva Ronni A.G., Tien Brenda Yin Qi, Kao Patrick Hsien Neng, Celik Cenk, Tan Ai Zhu Casandra, Ismail Muhammad Hafiz, Hu Guangan, Chong Kelvin Kian Long Chong, Thibault Guillaume, Chen Jianzhu, Kline Kimberly A.. 2025. Enterococcus faecalis -derived lactic acid facilitates persistent and polymicrobial wound infections by suppressing macrophage activation. bioRxiv. doi:10.1101/2025.01.31.635924 41605216

