# Supplementary material for: Genome-wide analysis of Enterococcus faecalis genes that facilitate interspecies competition with Lactobacillus crispatus
Source: J Bacteriol. 2025 Feb 4;207(3):e00438-24. doi: 10.1128/jb.00438-24 (PMC11925238; doi:10.1128/jb.00438-24)
Supplement: Supplemental figures — Fig. S1 to S6. [file jb.00438-24-s0001.docx]

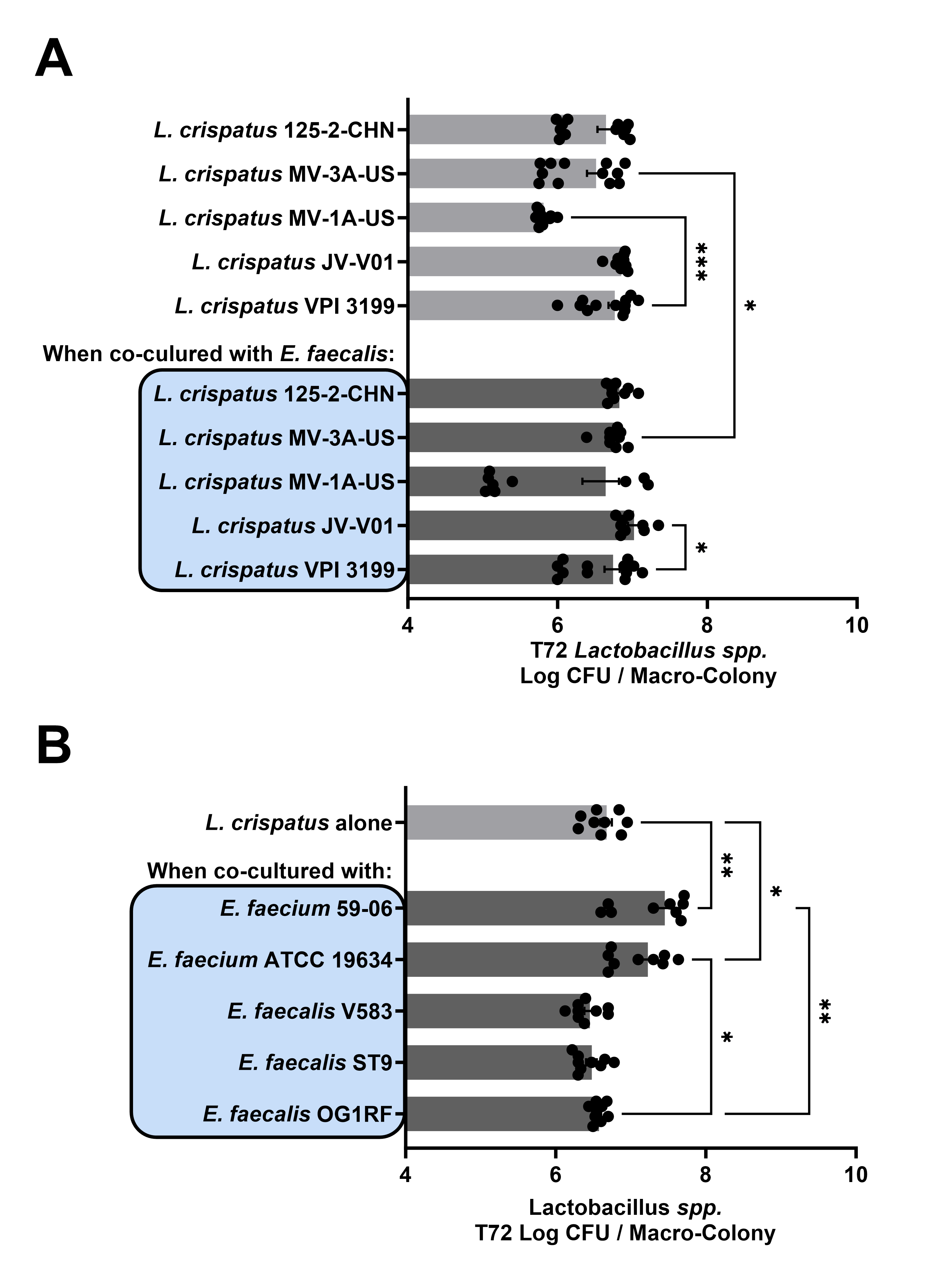


**Fig S1. *L. crispatus* antagonism of enterococci is conserved at the species level.** Colony-forming units (CFU) recovered from *L. crispatus* clinical strains grown either as single-species macro-colony biofilm, or as dual-species (with *E. faecalis* OG1RF) macro-colony biofilm for 72 hrs (A). Colony-forming units (CFU) recovered from *L. crispatus* VPI 3199 grown either as single-species macro-colony biofilm, or as dual-species (with clinical enterococcal strains) macro-colony biofilm for 72 hrs (B). Data points represent 9-12 biological replicates, collated from at least three repeated experiments. Statistical analysis was performed using Brown-Forsythe ANOVA test with Welch’s correction. Error bars represent standard error of the mean. * *p* ≤ 0.05, ** *p* ≤ 0.01, *** *p* ≤ 0.001.


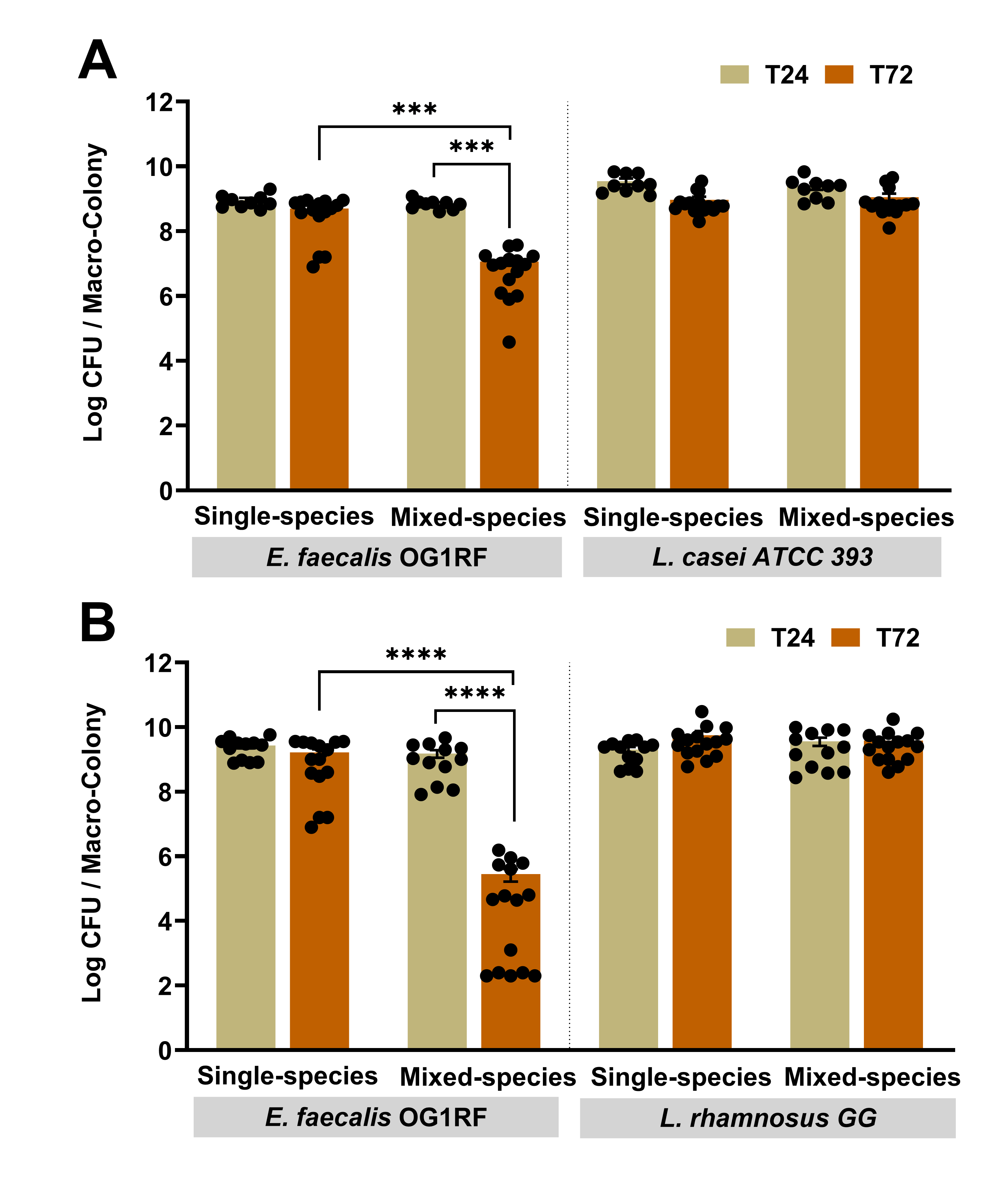


**Fig S2. *L. rhamnosus* and *L. casei* are antagonists.** Colony-forming units (CFU) recovered from *E. faecalis* OG1RF, *L. casei*, and *L. rhamnosus* respectively when grown either as single-species macro-colony biofilm, or as dual-species macro-colony biofilms (with *L. casei* (A) or with *L. rhamnosus* (B)) at 24 and 72 hrs. Data points represent 9-12 biological replicates, collated from at least three repeated experiments. Statistical analysis was performed using Brown-Forsythe ANOVA test with Welch’s correction. Error bars represent standard error of the mean. * *p* ≤ 0.05, ** *p* ≤ 0.01, *** *p* ≤ 0.001, **** *p* ≤ 0.0001.


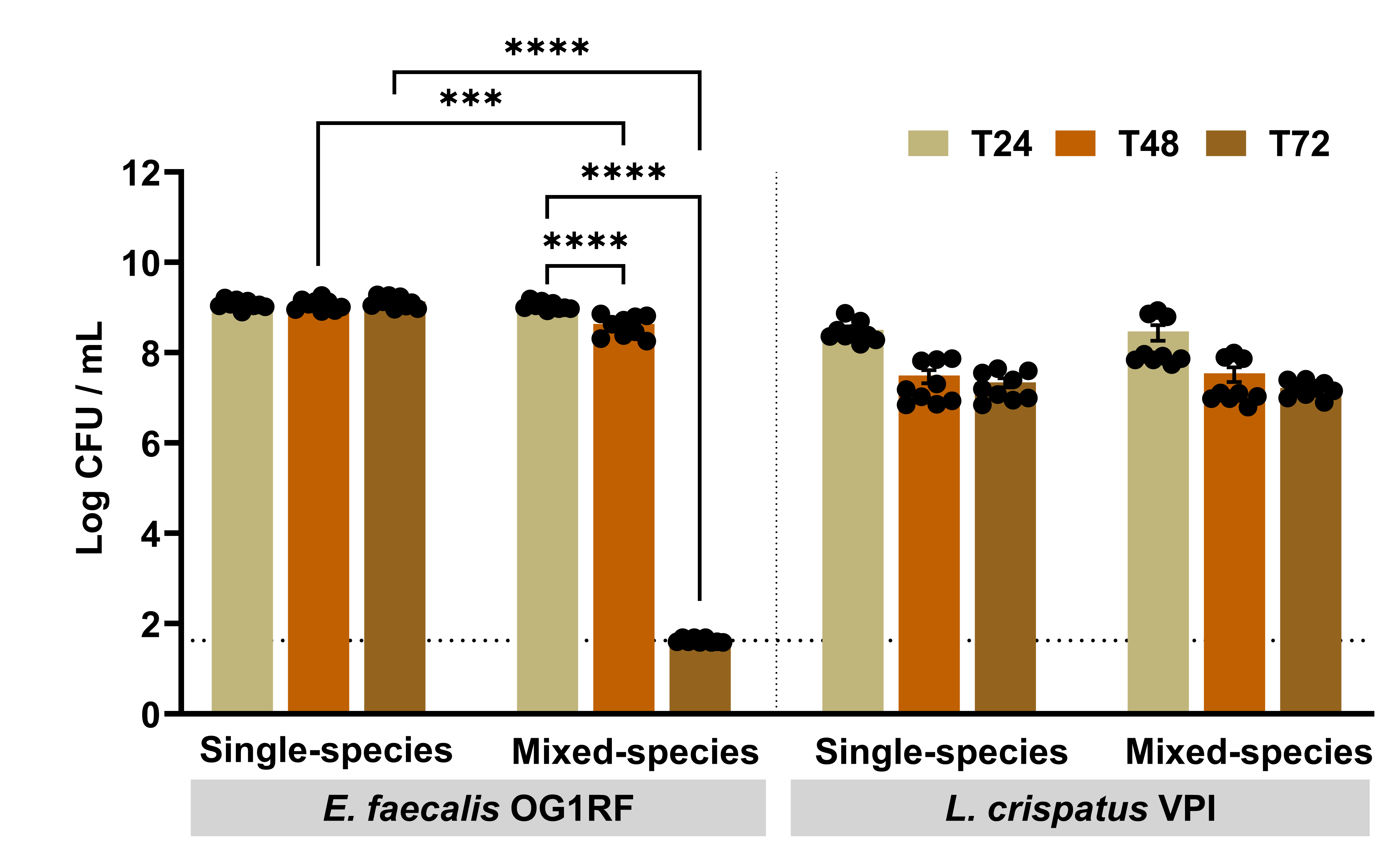


**Fig S3. *L. crispatus* biofilm antagonistic activity is strengthened when in broth-based assays.** Colony-forming units (CFU) recovered from *E. faecalis* OG1RF (A) and *L. crispatus* VPI 3199 (B) when grown statically either as single-species biofilm, or as dual-species (with *E. faecalis* OG1RF) biofilm for up to 72 hrs using tissue culture plates. Data points represent 9 biological replicates, collated from three repeated experiments. Statistical analysis was performed using Brown-Forsythe ANOVA test with Welch’s correction. Dotted line represents limit of detection; CFU of 42. Error bars represent standard error of the mean. **** *p* ≤ 0.0001.

**Fig S4. *L. crispatus* eradicates mature (24hrs) *E. faecalis* biofilm.** Assessment of *E. faecalis* OG1RF and *L. crispatus* VPI 3199 ability to grow respectively, in 24 hrs mature (pre-formed) *L. crispatus* and *E. faecalis* single-species biofilms grown in MRS or MRS supplemented with 300 mM MOPS media, and compared to their single-species biofilm counterpart. Colony-forming units (CFU) recovered respectively from *E. faecalis* OG1RF and *L. crispatus* VPI 3199 growth after 24 hr (A, C) and 72 hr (B, D) on pre-formed biofilms, or as single-species biofilms. Data points represent 6 biological replicates, collated from two experiments. Dotted line indicate limit of detection; CFU<83. Statistical analysis was performed using Brown-Forsythe ANOVA test with Dunnett’s T3 multiple comparison test. Error bars represent standard error of the mean. * *p* ≤ 0.05, ** *p* ≤ 0.01, *** *p* ≤ 0.001, **** *p* ≤ 0.0001.


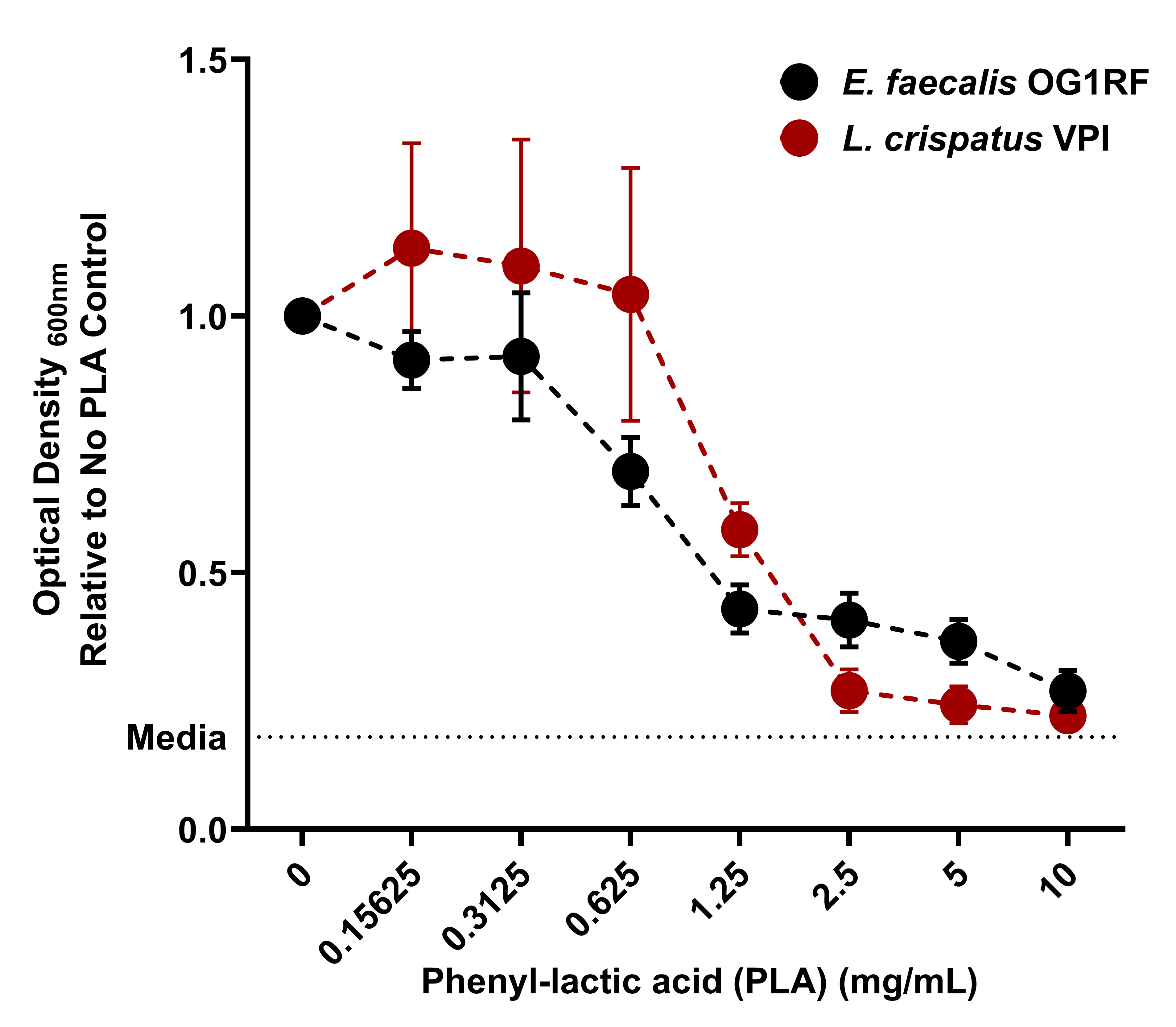


**Fig S5. Lactobacilli and *E. faecalis* have similar tolerance to lactobacilli-derived phenyl-lactic acid *in vitro*.** Growth of *E. faecalis* OG1RF and *L. crispatus* VPI 3199 in MRS media supplemented exogenously with increasing concentration of phenyl-lactic acid (PLA) after 24 hrs. Data points represent 3 biological replicates. Statistical analysis was performed using Wilcoxon matched pairs signed rank test. Error bars represent standard deviations (S.D). Dotted line represents optical density of the media (control).


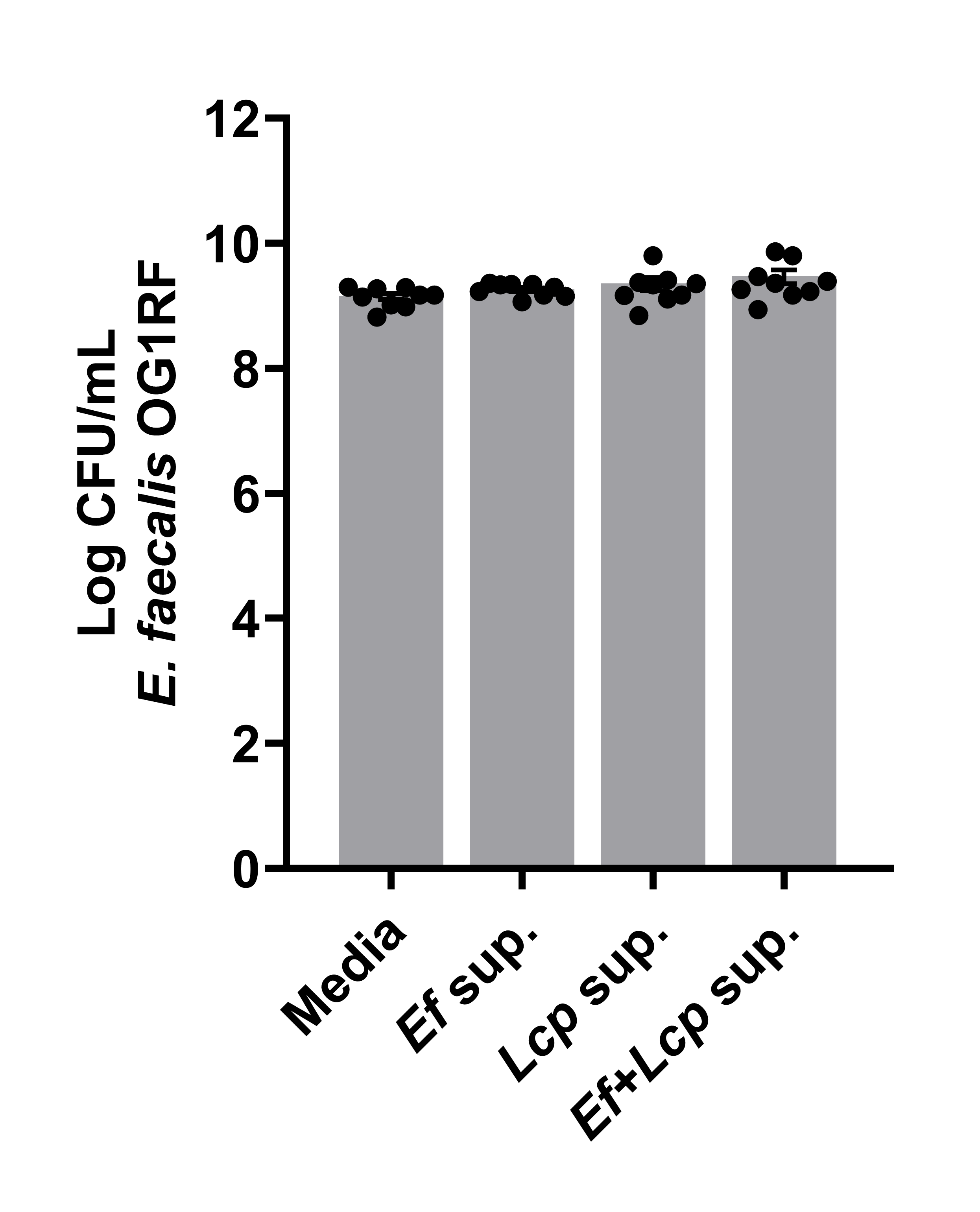


**Fig S6. Testing of biofilm supernatants for anti-enterococcal molecules by altering media ratios.** Colony-forming units (CFU) recovered from *E. faecalis* OG1RF growth after 24 hrs in MRS media mixed with 72 hrs cell-free biofilm supernatant isolated from single-species and dual-species biofilms, that was mixed at 90:10 ratio with fresh MRS, then adjusting media to final pH of 6.5. Data points represent 9-12 biological replicates, collated from at least three repeated experiments. Statistical analysis was performed using Brown-Forsythe ANOVA test with Welch’s correction. Error bars represent standard error of the mean.
